# Variants in the *CETP* gene affect levels of HDL cholesterol by reducing the amount, and not the specific lipid transfer activity, of secreted CETP

**DOI:** 10.1371/journal.pone.0294764

**Published:** 2023-12-01

**Authors:** Åsa Schawlann Ølnes, Marianne Teigen, Jon K. Laerdahl, Trond P. Leren, Thea Bismo Strøm, Katrine Bjune

**Affiliations:** 1 Unit for Cardiac and Cardiovascular Genetics, Oslo University Hospital, Oslo, Norway; 2 Department of Microbiology, Oslo University Hospital, Oslo, Norway; 3 Department of Informatics, ELIXIR Norway, University of Oslo, Oslo, Norway; Czestochowa University of Technology: Politechnika Czestochowska, POLAND

## Abstract

**Background:**

Cholesteryl ester transfer protein (CETP) transfers cholesteryl esters in plasma from high density lipoprotein (HDL) to very low density lipoprotein and low density lipoprotein. Loss-of-function variants in the *CETP* gene cause elevated levels of HDL cholesterol. In this study, we have determined the functional consequences of 24 missense variants in the *CETP* gene. The 24 missense variants studied were the ones reported in the Human Gene Mutation Database and in the literature to affect HDL cholesterol levels, as well as two novel variants identified at the Unit for Cardiac and Cardiovascular Genetics, Oslo University Hospital in subjects with hyperalphalipoproteinemia.

**Methods:**

HEK293 cells were transiently transfected with mutant CETP plasmids. The amounts of CETP protein in lysates and media were determined by Western blot analysis, and the lipid transfer activities of the CETP variants were determined by a fluorescence-based assay.

**Results:**

Four of the CETP variants were not secreted. Five of the variants were secreted less than 15% compared to the WT-CETP, while the other 15 variants were secreted in varying amounts. There was a linear relationship between the levels of secreted protein and the lipid transfer activities (r = 0.96, p<0.001). Thus, the secreted variants had similar specific lipid transfer activities.

**Conclusion:**

The effect of the 24 missense variants in the *CETP* gene on the lipid transfer activity was mediated predominantly by their impact on the secretion of the CETP protein. The four variants that prevented CETP secretion cause autosomal dominant hyperalphalipoproteinemia. The five variants that markedly reduced secretion of the respective variants cause mild hyperalphalipoproteinemia. The majority of the remaining 15 variants had minor effects on the secretion of CETP, and are considered neutral genetic variants.

## Introduction

High density lipoprotein (HDL) transports cholesterol from peripheral cells to the liver for excretion through the bile in a process referred to as reverse cholesterol transport [1, 2]. As part of this process, cholesteryl esters are transferred from HDL to very low density lipoprotein (VLDL) and low density lipoprotein (LDL) by the action of cholesteryl ester transfer protein (CETP) [3–6]. The role of CETP in the transfer of cholesteryl esters from HDL is illustrated by the markedly increased levels of HDL cholesterol in subjects lacking CETP [7].

CETP is a 493-residue protein with a 17-residue signal peptide [8]. The CETP gene spans 25 kb on the long arm of chromosome 16 and consists of 16 exons [9, 10]. CETP belongs to the tubular lipid-binding (TULIP) superfamily, as one of 14 members of the bactericidal/permeability-increasing protein-like TULIP proteins [11]. Members of this family are built from two tandemly repeated TULIP domains [11]. They are water soluble with a large hydrophobic cavity. A crystal structure has revealed that CETP is shaped like a “boomerang” with a 60 Å long hydrophobic tunnel traversing the core of the protein and having to distinct openings [12]. Within this tunnel, there were two cholesteryl ester molecules and two phospholipid molecules. The cholesteryl esters were buried in the middle of the tunnel, separated by a narrow part referred to as the neck, while the two phospholipids were located with their hydrophilic head groups at the two tunnel openings [12]. One or both cholesteryl ester molecules within the cavity can most likely be replaced by a triglyceride molecule or other lipids during lipid transport [12]. We are not aware of any experimental studies that have found CETP without bound lipids, and molecular dynamics simulations strongly suggest that the CETP protein structure is unstable and collapses without interior lipids [13].

Even though the lipid transfer activity of CETP has been well established, controversies exist regarding the molecular mechanism by which CETP transfers cholesteryl esters and triglycerides between lipoproteins. There are two main models for this transfer. One is that CETP may form a ternary complex between HDL and VLDL/LDL and the other is that CETP sequentially interacts with HDL and VLDL/LDL in a lipid shuttling mechanism [14].

One strategy to provide information about the structure-function relationship of CETP is to identify the mechanism by which the naturally occurring variants in the *CETP* gene affect the function of CETP. In this respect, there are reports in the Human Gene Mutation Database (HGMD) [15] that variants in the *CETP* gene may act as loss-of-function variants leading to high HDL cholesterol levels, and also that variants may act as gain-of-function variants leading to low HDL cholesterol levels. However, functional studies to determine the effects of missense variants on CETP activity are often lacking. In this study, we have selected 24 naturally occurring missense variants in the *CETP* gene that have been reported to affect levels of HDL cholesterol, to study how they affect the lipid transfer activity.

## Materials and methods

### Reagents

Trimethylamine *N*-oxide (TMAO), 4-phenylbutyrate (4-PBA), glycerol, dimethyl sulfoxide (DMSO), and 1,4-dithiothreitol (DTT) were obtained from Sigma-Aldrich Corp. (St. Louis, MO). Endoglycosidase H (endo H) was from New England Biolabs (Ipswich, MA). All other reagents were of analytical grade.

### Genetic variants

The variants selected for this study were the 20 CETP missense variants reported in HGMD [15] as of the fall of 2019, to affect the levels of HDL cholesterol (Table 1). In addition, two missense variants (R154W and Y378C) associated with increased HDL cholesterol levels [16], and two novel variants (R175Q and A291G) identified at the Unit for Cardiac and Cardiovascular Genetics, Oslo University Hospital in subjects with hyperalphalipoproteinemia, were included (Table 1). The four CETP variants A45V, G331S, V340I, and E420K (Table 1) were selected from the Infinium CoreExome-24 Kit (20039222; Illumina, San Diego, CA), which is being used for genome-wide association studies. To our knowledge, these four variants have not been associated with abnormal HDL cholesterol levels, and they were therefore included to illustrate the normal variation in CETP activity. Assessment of the pathogenicity of the variants was based on the guidelines from The American College of Medical Genetics and Genomics (ACMG) and The Association for Molecular Pathology [17]. Codon #1 of the *CETP* gene (NM_000078.3) was the ATG initiation codon, and nucleotide #1 was A of the ATG initiation codon. Protein structure illustration of variant residues was generated with PyMOL (Molecular Graphics System, version 2.2, Schrödinger, LLC).

**Table 1:** Missense variants in the *CETP* gene subjected to functional characterization.

Variant			Phenotype*	HGMD^#^ class	*In silico* predictions			gnomAD^&^		
Protein level	Nucleotide level	rsID			SIFT	MutationTaster	PolyPhen2		ACMG^§^ class	Reference
**Variants not reported to affect HDL cholesterol levels**										
A45V	c.134C>T	rs200956328	-	-	Tolerated	Benign	Benign	1/18947 (NFE)	3	-
G331S	c.991G>A	rs5881	-	-	Tolerated	Benign	Benign	1/207 (EA)	2	-
V340I	c.1018G>C	rs141310739	-	-	Tolerated	Benign	Benign	1/7653 (SA)	2	-
E420K	c.1258G>A	rs202026395	-	-	Tolerated	Benign	Benign	1/37908 (NFE)	3	-
**Variants reported to affect HDL cholesterol levels**										
V6D	c.17T>A	rs34119551	High HDLC	DM	Tolerated	Benign	Benign	1/461 (A)	4	[18]
A15G	c.44C>G	rs34065661	High/Low HDLC	DP	Tolerated	Benign	Benign	1/14 (A)	2	[19]
T61M	c.182C>T	rs142464301	Low HDLC	DM	Tolerated	Deleterious	Possibly Damaging	1/3228 (ENF)	3	[20]
D131N	c.391G>A	rs775508511	High HDLC	DM?	Not Tolerated	Benign	Possibly Damaging	1/16248 (ENF)	3	[21]
R154W	c.460C>T	rs184615182	High HDLC	-	Tolerated	Benign	Benign	1/35 (A)	2	[16]
R154Q	c.461G>A	rs34716057	High HDLC	DM	Tolerated	Deleterious	Benign	1/3988 (EA)	3	[20]
L168P	c.503T>C	-	High HDLC	DM	Tolerated	Deleterious	Probably Damaging	-	5	[22]
R175Q	c.524G>A	rs145690607	High HDLC	-	Tolerated	Benign	Possibly Damaging	1/3807 (SA)	3	Novel^1^
S221R	c.663C>A	rs201438792	Low HDLC	DM	Tolerated	Benign	Benign	1/48 (SA)	2	[20]
G251V	c.752 G>T	rs144460063	Low HDLC	DM	Not Tolerated	Benign	Probably Damaging	1/6441 (ENF)	3	[21]
S268L	c.803C>T	rs747307952	High HDLC	DM	Tolerated	Deleterious	Benign	1/1932 (EF)	2	[21]
L278R	c.833T>G	rs755528414	High HDLC	DM	Not Tolerated	Deleterious	Probably Damaging	1/6130 (EA)	5	[23]
A291G	c.872C>G	rs200375648	High HDLC	-	Tolerated	Deleterious	Benign	-	3	Novel^2^
A291D	c.872C>A	Novel	High HDLC	DM	Not Tolerated	Deleterious	Probably Damaging	1/2298 (EA)	3	[24]
R299C	c.895C>T	rs142459781	High HDLC	DM	Not Tolerated	Deleterious	Probably Damaging	1/3119 (A)	3	[22]
L313Q	c.938T>A	-	High HDLC	FP	Not Tolerated	Benign	Probably Damaging	-	3	[25]
E314K	c.940G>A	rs140547417	High HDLC	DM	Tolerated	Deleterious	Benign	1/1195 (ENF)	3	[20]
S349Y	c.1046C>A	rs752298084	High HDLC	FP	Tolerated	Benign	Probably Damaging	1/577 (SA)	2	[26]
Y378C	c.1133A>G	rs36122917	High HDLC	-	Tolerated	Benign	Possibly Damaging	1/22751 (ENF)	3	[16]
A390P	c.1168G>C	rs5880	Low HDLC	FP	Not Tolerated	Benign	Probably Damaging	1/19 (ENF)	2	[27]
E443K	c.1327G>A	rs536221680	High HDLC	FP	Not Tolerated	Benign	Possibly Damaging	1/8647 (L)	3	[28]
S452G	c.1354A>G	rs1479890768	Low HDLC	DM	Tolerated	Benign	Probably Damaging	1/32285 (ENF)	3	[20]
D459G	c.1376A>G	rs2303790	High HDLC	DM	Not Tolerated	Benign	Possibly Damaging	1/30 (EA)	4	[29]
R468Q	c.1403G>A	rs1800777	High HDLC	FP	Tolerated	Benign	Benign	1/9 (L)	1	[30]

*Phenotype associated with the individual variant: HDLC: HDL cholesterol level

^#^Human Gene Mutation Database (HGMD) class: DM: Disease-causing mutation, DM?: Disease-causing mutation?, DP: Disease-associated phenotype, FP: *in vivo* or *in vitro* functional polymorphism, -: Not listed in HGMD.

^&^Allele frequencies from Genome Aggregation Database (gnomAD). The highest allele frequencies among the populations: African (A), Latino (L), East Asian (EA), South Asian (SA), European Non-Finnish (ENF) or European Finnish (EF) are shown.

^§^Classification of pathogenicity was performed according to the guidelines from The American College of Medical Genetics and Genomics and The Association for Molecular Pathology (ACMG). Class 1: Not pathogenic, Class 2: Unlikely pathogenic, Class 3: Unknown pathogenicity, Class 4: Likely pathogenic, Class 5: Pathogenic.

Novel^1^: Variant R175Q was identified at Unit for Cardiac and Cardiovascular Genetics, Oslo University Hospital in a patient with an HDL cholesterol value of 4.5 mmol/L.

Novel^2^: Variant A291G identified at Unit for Cardiac and Cardiovascular Genetics, Oslo University Hospital in a patient with an HDL cholesterol value of 4,2 mmol/L.

Missense variants in the *CETP* gene not associated with altered HDL cholesterol levels used as controls, and missense variants that have been reported to affect HDL cholesterol levels are shown. The variants are annotated with respect to their effects at the protein level and at the nucleotide level. The rsIDs for the specific single nucleotide polymorphisms are shown. The reported phenotypic effects of the variants on HDL cholesterol levels, as well as the classifications made by HGMD for variants reported in that database are shown. Also shown are the *in silico* predictions of pathogenicity generated by Sorting Intolerant from Tolerant (SIFT) [31], MutationTaster [32] and Polymorphism Phenotyping v2 (PolyPhen2) [33]. Allele frequencies of the variants obtained from the Genome Aggregation Database (https://gnomad.broadinstitute.org/) (gnomAD), and pathogenicities of the variants assessed according to the ACMG guidelines are shown.

### Plasmids and transfections

To generate a CETP plasmid, RNA was isolated from immortalized human hepatocytes (a kind gift from Dr. Philippe Costet, Paul Sabatier University, Toulouse, France) using the EB Monarch Total RNA Miniprep Kit (T2010S; New England Biolabs, Ipswich, MA) according to the manufacturer’s instructions. cDNA was synthesized from 1 μg RNA using AffinityScript QPCR cDNA Synthesis Kit (600559; Agilent Technologies, Santa Clara, CA), and CETP cDNA was amplified from 900 ng total cDNA using the Q5® High-Fidelity DNA Polymerase Kit (M0491S; New England Biolabs, Ipswich, MA). Amplification oligonucleotides are listed in S1 Table. The polymerase chain reaction (PCR) product was purified using Monarch® DNA Gel Extraction Kit (T1020S; New England Biolabs, Ipswich, MA). T-overhang was added to the PCR product using OneTaq DNA Polymerase (M0480S; New England Biolabs, Ipswich, MA), and the PCR product was TA-cloned into a pcDNA3.1 V5/His TOPO TA vector (K480001; Invitrogen, Waltham, MA) according to the manufacturer’s instructions. The resulting wild-type (WT) CETP plasmid contained p.Ile422 (c.1264G>A), which is considered a neutral genetic variant with an allele frequency of 0.62 according to the Genome Aggregation Database (gnomAD) (https://gnomad.broadinstitute.org/). The other frequent variant at this residue is p.Val422, and the lipid transfer activity, synthesis, and intracellular transport of both variants (WT-CETP and I422V) are tested and compared in S1 Fig, with no significant difference. Assessment of the vector-based tags on the activity of the CETP protein showed that the construct containing both V5- and His-tag had a lower lipid transfer activity than WT-CETP without the latter. A stop codon was therefore inserted between the two tags to obtain the highest activity baseline for the assay. The resulting plasmid pcDNA3.1-WT-CETP-V5 was used as a template to generate mutant CETP plasmids. Mutageneses were carried out using One Shot™ TOP10 Chemically Competent *E*. *coli* (C404010; Invitrogen, Waltham, MA) and QuikChange II XL Mutagenesis Kit (200522–5; Agilent Technologies, Santa Clara, CA) according to the manufacturer’s instructions. The sequences of the mutageneses oligonucleotides are listed in S1 Table. Plasmids were isolated using NucleoSnap Plasmid Midi kit (740494.50; Macherey-Nagel GmbH & Co. KG, Düren, Germany). The integrity of the plasmids was verified by Sanger sequencing. HEK293 cells (European Collection of Authenticated Cell Cultures, Wiltshire, UK) were reverse transfected at 30% confluence for 24 h using FuGENE® HD (E2312; Promega, Madison, WI) according to the manufacturer’s instructions.

### Cell cultures

HEK293 cells were cultured in Modified Eagle’s medium (SH30244.01; Global Life Sciences Solutions USA, Marlborough, MA) containing 10% fetal bovine serum, 2 mM L-glutamine, 50 U/mL penicillin, 50 μg/mL streptomycin and non-essential amino acids. The cells were grown on Nunclon plates (159910; Thermo Fisher Scientific, Waltham, MA). Opti-MEM medium without phenol red (11058–021; Gibco Life Technologies, Paisley, UK) supplemented with 50 U/mL penicillin and 50 μg/mL streptomycin was used as minimal culture medium.

### Preparation of cell lysates and media

Twenty-four hours after transfection, HEK293 cells were washed with phosphate-buffered saline and the medium was replaced with minimal medium for a 16 h incubation. The medium was collected and cell debris was removed by centrifugation (2655 *g* for 5 min at 22°C). Cells were lysed by scraping in an in-house lysis buffer (1% Triton X-100, 150 mM NaCl and 10 mM Tris/HCl (pH 7.4)) containing Complete™ Protease Inhibitor Cocktail (11836170001; Roche Diagnostics, Basel, Switzerland), frozen (30 min at -80°C) and cleared by centrifugation (20817 *g* for 15 min at 4°C). The protein concentration of the lysate was determined using the Pierce BCA Protein Assay Kit (23227; Thermo Fisher Scientific, Waltham, MA). 15 μg of cell lysate and 30 μL of culture medium were used for Western blot analyses.

### Western blot analysis

Cell lysates and media were subjected to sodium dodecyl sulfate-polyacrylamide gel electrophoresis on 4–20% Tris-HCl Criterion™ TGX™ Precast Gels (5671094; BioRad Laboratories, Inc., Hercules, CA) for 23 min at 300 V. Lysates and media were blotted onto Immuno-Blot Polyvinylidene difluoride membranes (1704159; BioRad Laboratories, Inc., Hercules, CA) using the Trans-Blot Turbo Transfer System (1704155EDU; BioRad Laboratories, Inc., Hercules, CA). Color Prestained Protein Standard Broad Range (P7719S; New England BioLabs, Ipswich, MA) was used as a molecular weight marker. Non-specific binding sites were blocked using 5% Blotting Grade Blocker Non-Fat Dry Milk (9999S; Cell Signaling Technology, Danvers, MA) for 1 h at 22°C. A horseradish peroxidase-conjugated mouse anti-V5 antibody (R961-25; Invitrogen, Waltham, MA) was used to detect V5-tagged CETP. β-actin was used as a loading control for lysates and was detected by a rabbit anti-β-actin antibody (ab213262; Abcam, Cambridge, UK). An anti-rabbit IgG conjugated with horseradish peroxidase (7074S, Cell Signaling Technology, Danvers, MA) was used as the secondary antibody. Bands were visualized by SuperSignal West Dura Extended Duration Substrate (34076X4; Thermo Fisher Scientific, Waltham, MA), and chemiluminescence detected on a ChemiDoc Touch Imaging System (1708370; BioRad Laboratories, Inc., Hercules, CA). Blots were analyzed with Image Lab V5.2.1 (BioRad Laboratories, Inc., Hercules, CA). The molecular weight of WT-CETP was 72 kDa in lysate and 74 kDa [7] in the medium. To account for the number of cells in the lysate secreting CETP, values from the quantitated Western blots of media were corrected for the protein concentration of the lysate of the respective sample.

### Assay for measuring lipid transfer activity of CETP

Lipid transfer activity of the different CETP variants was measured in 10 μL of media, using the CETP Activity Assay Kit II (ab196995; Abcam, Cambridge, UK) according to the manufacturer’s instructions. In brief, samples were read on a plate reader every 10 min for 1 h, and every 30 min the following 3 h. A trend line was generated from this data, and two sample reading times were used to calculate the lipid transfer activity for each sample. There was a linear correlation between the concentration of CETP protein and lipid transfer activity (S2 Fig). To account for the number of cells in the lysate secreting CETP, values from the measured lipid transfer activity were corrected for the protein concentration of the lysate of the respective sample. Values for specific lipid transfer activity were obtained by dividing the lipid transfer activity by the amount of CETP protein detected in the media as determined by Western blot analysis.

### Quantitative real-time PCR

AffinityScript QPCR cDNA Synthesis Kit (600559; Agilent Technologies, Santa Clara, CA) was used to synthesize cDNA from RNA isolated from transiently transfected HEK293 cells. cDNA was subjected to quantitative real-time PCR (qPCR) in triplicates using Luna Universal Probe qPCR Master Mix (M3004E; New England BioLabs, Ipswich, MA), and run on Aria Mx Real Time PCR System (G8830A; Agilent Technologies, Santa Clara, CA). The qPCR cycling conditions included a 1 min initial denaturation step at 95°C, followed by 40 cycles of 15 s denaturation at 95°C and 30 s extension at 60°C. Probes for qPCR were PrimeTime Predesigned qPCR Assay primers (Integrated DNA Technologies, Coralville, IA) for CETP (Hs.PT.58.14760793) and glyceraldehyde-3-phosphate dehydrogenase (GAPDH) (Hs.PT.39a.22214836). Using the 2^−ΔΔCt^ method [34], the level of target mRNA was determined and normalized to the housekeeping gene GAPDH.

### RT-PCR analysis of XBP1 RNA splicing to identify ER stress

RNA isolated from transiently transfected HEK293 cells was subjected to reverse transcriptase-PCR (RT-PCR) using OneStep RT-PCR kit (210212; Qiagen, Hilden, Germany) according to the manufacturer’s instructions. RT-PCR cycling conditions included a 15 min polymerase activation step at 95°C, 40 cycles of 1 min denaturation at 94°C, 1 min annealing at 60°C and 1 min extension at 72°C, followed by a final extension for 10 min at 72°C. X-box binding protein 1 (XBP1) mRNA was amplified using the forward primer: 5’-CTGGAAAGCAAGTGGTAGA-3’ and the reverse primer: 5’-CTGGGTCCTTCTGGGTAGAC-3’. RT-PCR products were stained with GelRed Nucleic Acid Stain (41003; Biotium Inc., Fremont, CA), and analyzed by electrophoresis at 4°C on a 2% Metaphor agarose gel (50180; Cambrex, East Rutherford, NJ) for 7 h at 50 V. TrackIT™ 100 bp DNA Ladder (10488058; Thermo Fisher Scientific, Waltham, MA) was used as a DNA sizing marker. The gel was imaged using a ChemiDoc Touch Imaging System (1708370; BioRad Laboratories, Inc., Hercules, CA). RNA isolateed from transiently transfected HEK293 cells incubated with 5 mM DTT overnight was used as a positive control [35].

### Statistical analysis

Data are presented as mean values and standard deviations (SD) unless otherwise noted. A linear regression analysis with a two-tailed t-test of two samples assuming equal variances was used to determine the Pearson correlation coefficient and its associated p value. The p values for the lipid transfer activities, amounts of CETP in media and lysates, and relative CETP mRNA expression were calculated by performing an F-test to check for equal variances, followed by a two-tailed t-test with two samples assuming equal or unequal variance, depending on the results of the F-test. A significance level of 0.05 was used. Stata version 17.0 (StataCorp LLC, College Station, TX, USA) was used to perform all statistical analyses.

## Results

### Lipid transfer activity, synthesis, and intracellular transport of CETP variants

**Fig 1:**
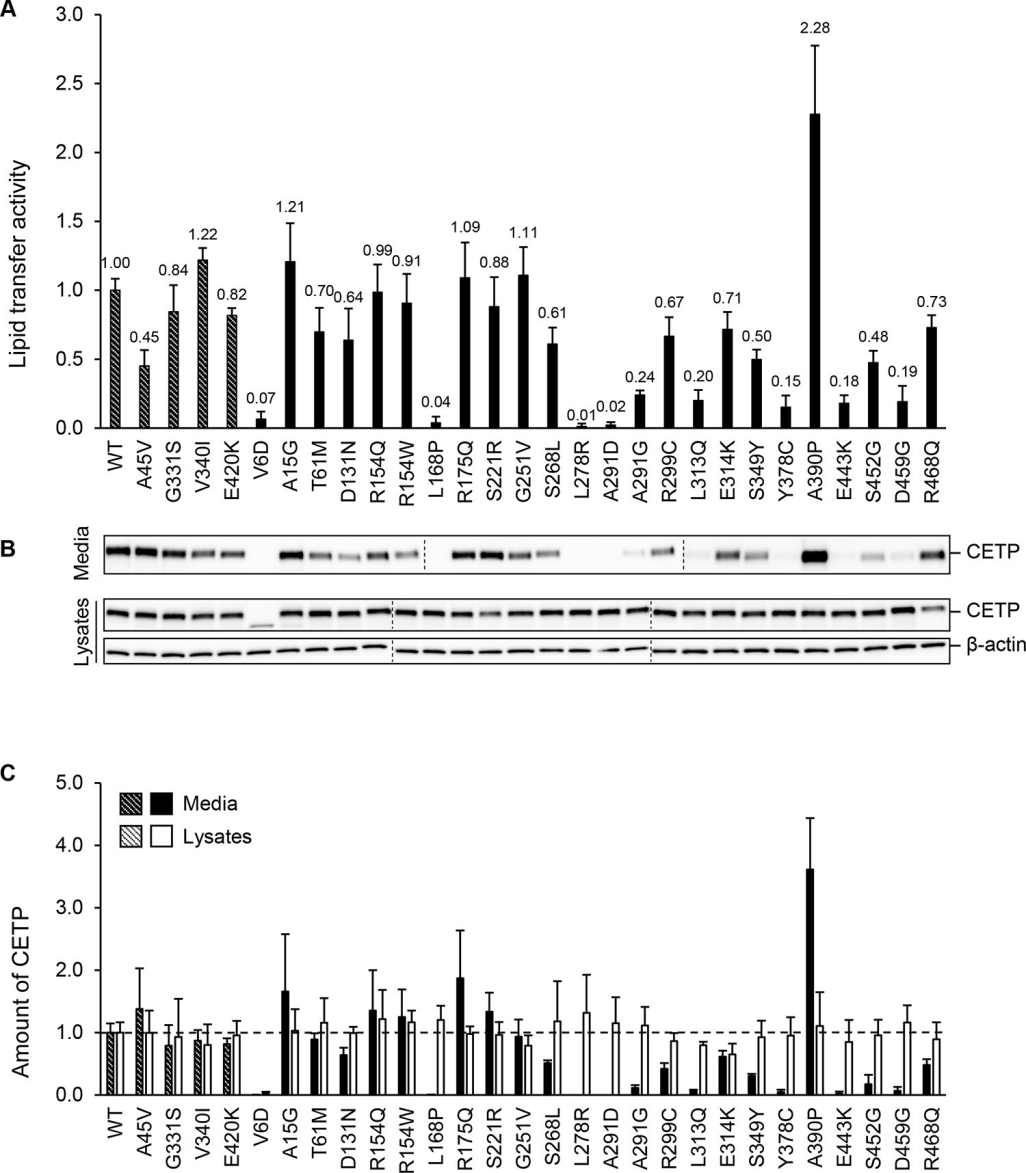
Lipid transfer activity, secretion, and synthesis of CETP missense variants. HEK293 cells were transiently transfected with the WT-CETP plasmid or the indicated CETP plasmids. (A) The lipid transfer activities in the media of transfected cells were determined. To account for the number of cells in the lysate secreting CETP, lipid transfer activity values were corrected for the protein concentration of lysates of the respective sample. The WT-CETP was assigned a value of 1.0. The values shown are the mean (±SD) of three separate experiments. For the lipid transfer activities, all variants except for G331S, A15G, D131N, R154Q, R154QW, R175Q, S221R, and G251V were significantly different from WT-CETP. (B) Culture media and lysates of transfected cells were subjected to Western blot analysis using an anti-V5 antibody directed against a C-terminal V5 tag. β-actin was used as a loading control for lysates. WT-CETP and the CETP variants A45V, G331S, V340I, and E420K (hatched symbols) were used as controls. The dashed vertical lines indicate that Fig 1B has been generated from more than one Western blot due to the large number of CETP variants studied. One representative experiment from three separate experiments is shown. (C) The mean values for the quantitated media and lysates from three separate experiments are shown as filled or closed bars, respectively. To account for the number of cells in the lysate secreting CETP, values of the amount of CETP in media were corrected for the protein concentration of lysates of the respective sample. For the amounts of CETP in media, all variants except for A45V, G331S, V340I, E420K, A15G, T61M, R154Q, R154W, R175Q, and G251V were significantly different from WT-CETP. Variants V6D and E314K were statistically different from WT-CETP for the level of CETP protein in lysates. All means, standard deviations, and p values for the lipid transfer activity, and amount of CETP protein in media and lysates for all CETP variants are listed in S2 Table.

To study how the 24 missense variants in the *CETP* gene affect the synthesis, intracellular transport, and lipid transfer activity of the protein, HEK293 cells were transiently transfected with each of the CETP plasmids (Fig 1A–1C). The levels of CETP protein in lysates and media were determined by Western blot analysis, and the lipid transfer activities of CETP in the media were determined by a fluorescence-based assay. The four variants A45V, G331S, V340I, and E420K are not reported to affect HDL levels, and cells transfected with plasmids encoding these variants were for that reason included to illustrate the normal variation in CETP activity. However, the lipid transfer activity of A45V of 45% compared to WT-CETP may question whether A45V is a true genetic variant affecting the serum HDL levels, and should be studied further.

Large differences in lipid transfer activities were found for the different CETP variants (Fig 1A). The same pattern was observed for the amounts of CETP protein in the media (Fig 1B and 1C). Four of the variants had lipid transfer activities close to zero and were either not found in the media, or found in markedly reduced levels. These were V6D, L168P, L278R, and A291D (Fig 1A–1C). Variants A291G, L313Q, Y378C, E443K, and D459G had low lipid transfer activities and were found in very low amounts in the media (Fig 1A–1C). The variant A390P had a two-fold increase in lipid transfer activity and was found in a higher amount in the media as compared to the WT-CETP (Fig 1A–1C). With one exception, V6D, similar levels of WT-CETP and the different CETP variants were found in lysates (Fig 1B and 1C). For the V6D variant, which affects the 17-residue signal peptide, no 72 kDa band representing mature CETP protein was found in the lysate. Instead, a shorter 64 kDa band was observed. Moreover, the intensity of this band was lower than that of the band observed for WT-CETP or the other CETP variants. A small amount of this 64 kDa band was also observed for A15G (Fig 1B).

### Studies of transfection efficiencies

The finding that similar amounts of the different CETP variants were found in lysates, indicates that there were no major differences in transfection efficiencies between the different plasmids. To confirm this notion, qPCR of CETP mRNA isolated from lysates of the transfected cells was performed (S3 Fig). Although some variation in the quantities of CETP mRNA was observed, we conclude that the different amounts of CETP variants in the media are not explained by differences in transfection efficiencies.

### The CETP V6D variant

**Fig 2:**
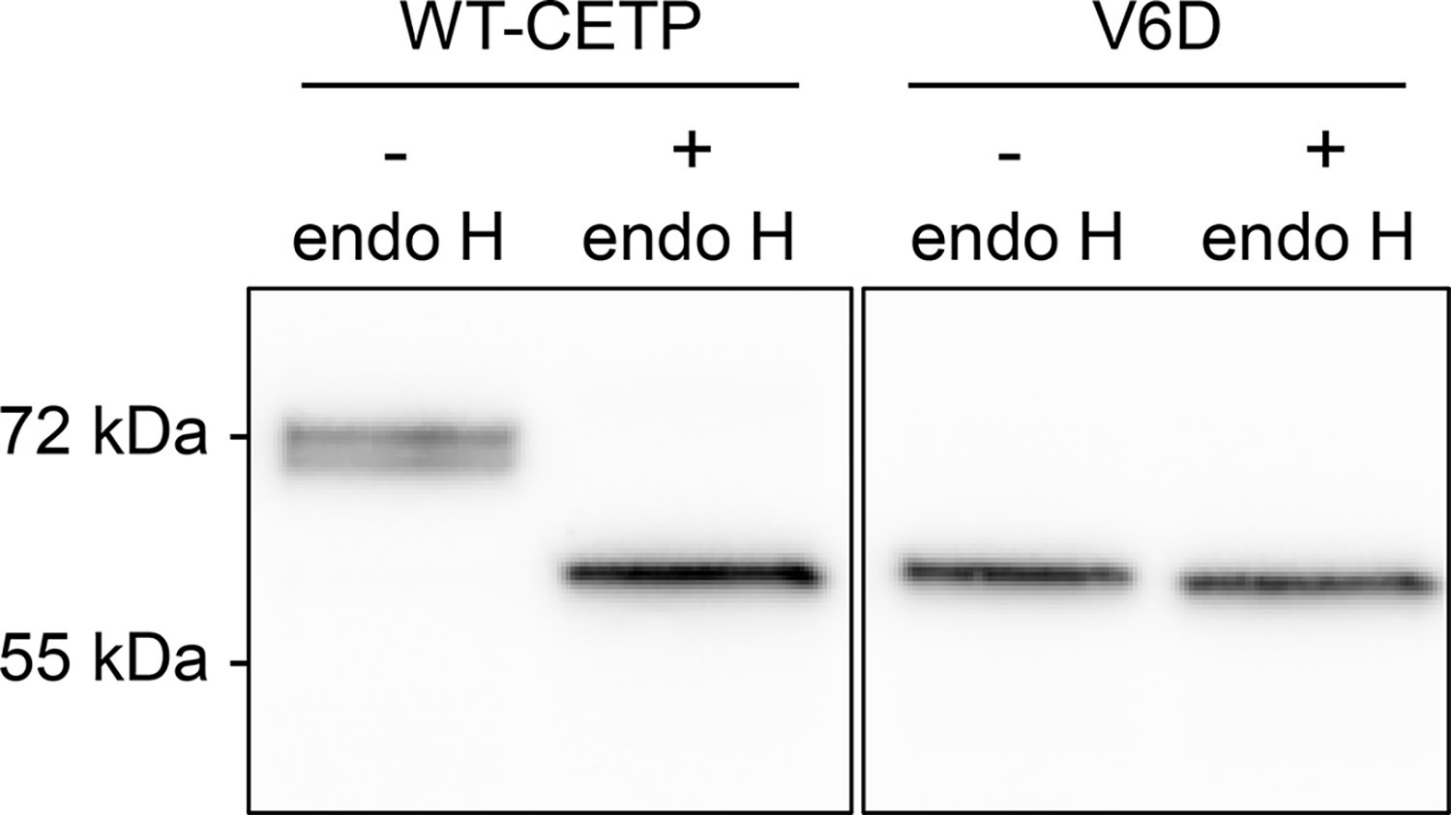
Sensitivity of the CETP V6D variant to be cleaved by endo H. HEK293 cells were transiently transfected with the WT-CETP plasmid or V6D plasmid. Lysates from the transfected cells were treated with H_2_O (-) or the deglycosylation enzyme endo H (500 U, 1 h) (+). Lysates were subjected to Western blot analysis using an anti-V5 antibody directed against a C-terminal V5 tag. One representative Western blot from three separate experiments is shown. The molecular weight markers representing 55 kDa and 72 kDa are indicated.

It is assumed that the reason for the lack of a 72 kDa V6D fragment in the lysate, is the failure of the mutant polypeptide to be translocated into the endoplasmic reticulum (ER). The underlying mechanism is the replacement of a hydrophobic valine with an acidic aspartate in the middle of the hydrophobic region of the signal peptide. To study this notion, the sensitivity of V6D to be cleaved by endo H was studied. Endo H cleaves N-linked oligosaccharides from the ER, whereas these oligosaccharides become insensitive to endo H cleavage after having been processed in the Golgi apparatus [36]. The failure of V6D to be cleaved by endo H is compatible with the notion that V6D is not translocated into the ER (Fig 2). Rather, the V6D variant is assumed to be located in the cytosol. The 8 kDa lower molecular weight then reflects the failure of this protein to undergo proper glycosylation in the ER. The observation that some of the 64 kDa band was found also for A15G, indicates that a proportion of this protein is not correctly translocated into the ER. Moreover, the finding that WT-CETP in the lysate was sensitive to cleavage by endo H indicates that the majority of WT-CETP in lysate has been processed in the ER (Fig 2). The two bands observed in WT-CETP without endo H likely represent different stages of CETP glycosylation. WT-CETP in the medium was not sensitive to cleavage by endo H (data not shown).

### Specific lipid transfer activity of CETP missense variants

**Fig 3:**
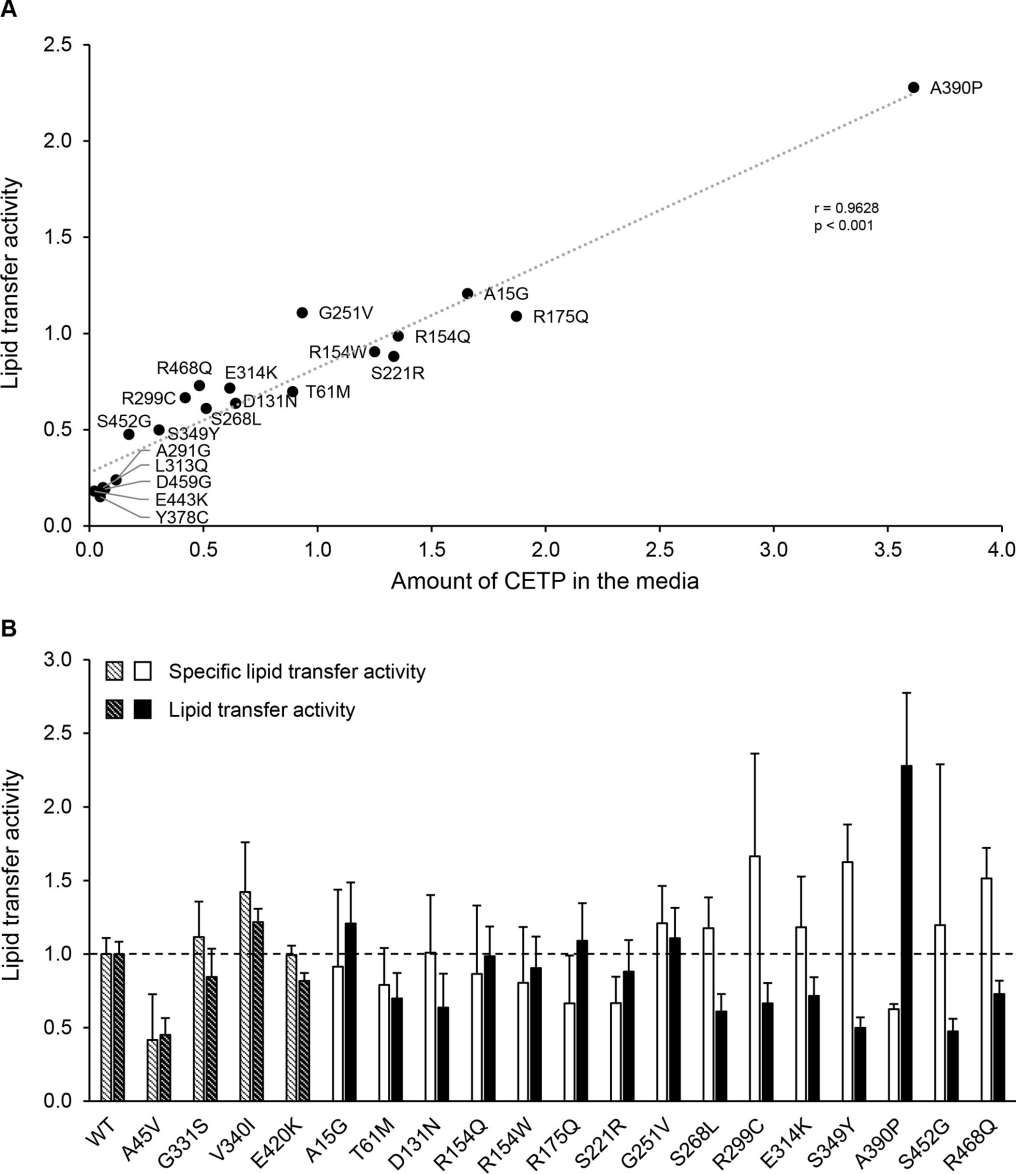
Lipid transfer activities compared to amounts of CETP protein in the media for the different CETP variants. (A) The relationship between the amounts of CETP in the media and the corresponding lipid transfer activities is shown. The levels of protein secreted for the 20 CETP variants from Fig 1B have been plotted against the respective lipid transfer activities obtained from Fig 1A. The Pearson correlation coefficient (r) and its associated p value (p) for the correlation between CETP mass and lipid transfer activity are shown. (B) Data for the amounts of CETP protein in the media of HEK293 cells transiently transfected with WT-CETP or CETP plasmids as well as the corresponding lipid transfer activities from Fig 1 are shown. To correct the lipid transfer activities obtained in Fig 1A (filled bars) for differences in the amounts of secreted CETP, the lipid transfer activities were divided by the levels of CETP in the media as determined by Western blot analyses. The corrected values are shown as specific lipid transfer activities (open bars). Only the 15 well-secreted variants of Class C (see main text) were included in the analysis. The lipid transfer activity for WT-CETP was assigned a value of 1.0. The values shown are the mean (±SD) of three separate experiments.

To study the functional consequences of the 24 CETP missense variants, the lipid transfer activity of the CETP variants in the media was determined (Fig 1A). As expected, the four non-secreted variants had lipid transfer activities that were close to zero. For the other 20 variants, differences in the lipid transfer activities could be due to functional differences or differences in the number of CETP protein secreted. The finding that there was a positive correlation between the levels of CETP in the media and the lipid transfer activity (r = 0.96, p<0.001) (Fig 3A) indicates that the major determinant for the lipid transfer activity of these 20 CETP missense variants, was the amount of secreted mutant protein. Therefore, data for specific lipid transfer activity was determined for the 15 variants that with accuracy could be quantified by Western blot analyses of media (Fig 3B). By specific lipid transfer activity, we refer to the activity of CETP relative to the amount of CETP protein in media. The specific lipid transfer activities for the 15 missense variants were similar to that of WT-CETP (Fig 3B). These data indicate that the lipid transfer activities of the 15 variants were not substantially different from that of WT-CETP.

**Fig 4:**
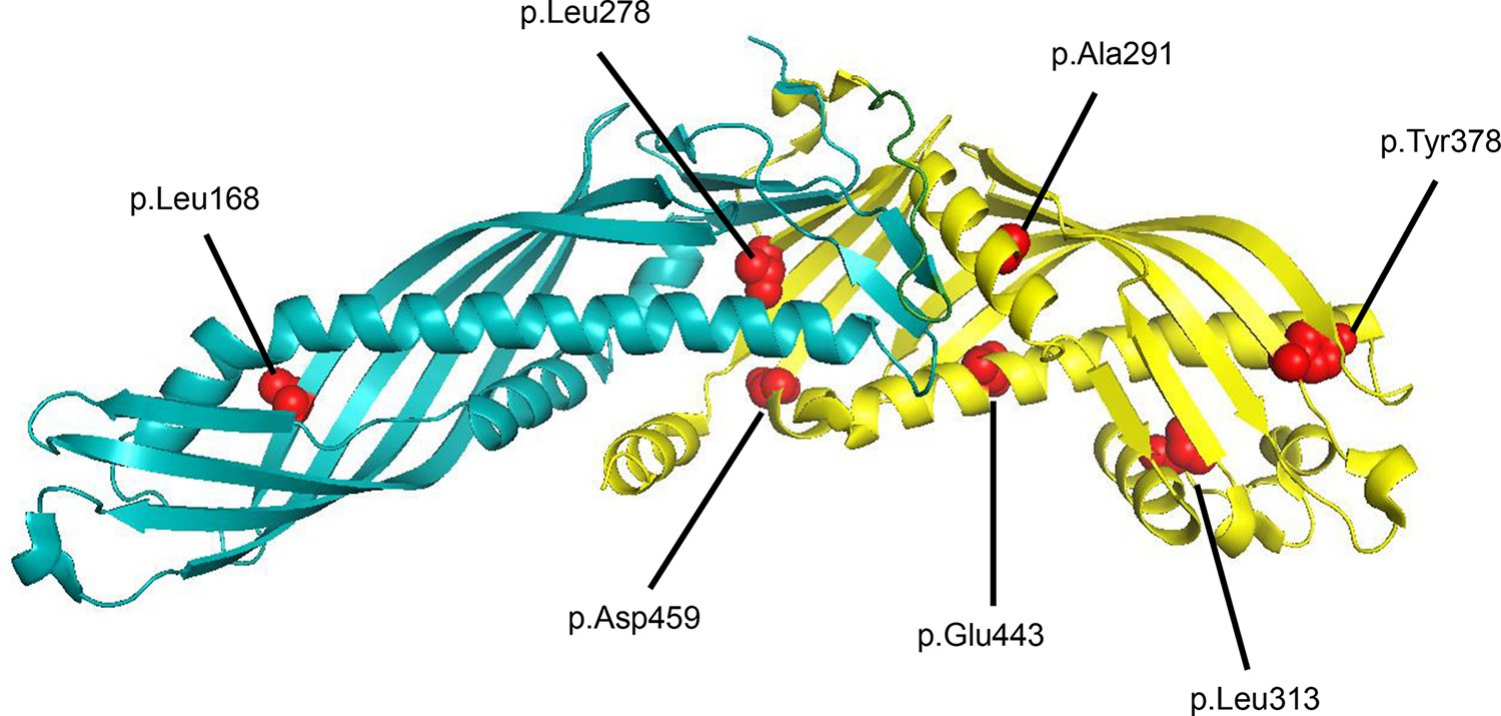
Location of residues involved in Class A and Class B variants. The structure of CETP is shown with the N-terminal TULIP domain (residues p.22-256) in cyan and the C-terminal TULIP domain (residues p.277-493) in yellow. Residues affected by Class A variants (p.Leu168, p.Leu278, p.Ala291) and Class B variants (p.Ala291, p.Leu313, p.Tyr378, p.Glu443, p.Asp459) are indicated. The structure is based on the Protein Data Bank structure 2OBD [13]. p.Val6 is located within the signal peptide of CETP and is thus not included in the crystal structure.

From the data in Figs 1A and 3A, it appears that the 24 CETP missense variants could be categorized into three classes. Class A consists of the four variants V6D, L168P, L278R, and A291D that were not secreted. Class B consists of the five variants A291G, L313Q, Y378C, E443K, and D459G which were secreted less than 15% compared to the WT-CETP. Class C consists of the remaining 15 variants that were rather well secreted, meaning that they had distinct bands on the Western blot of the media. The locations of residues involved in Class A and B are shown in Fig 4. The evolutionary conservation of all the mutated residues is shown in S4 Fig. It is worth noting that variants impacting highly conserved residues are more likely to be pathogenic compared to those affecting poorly conserved residues.

### Analysis of ER stress generated by CETP missense variants that are not secreted

**Fig 5:**
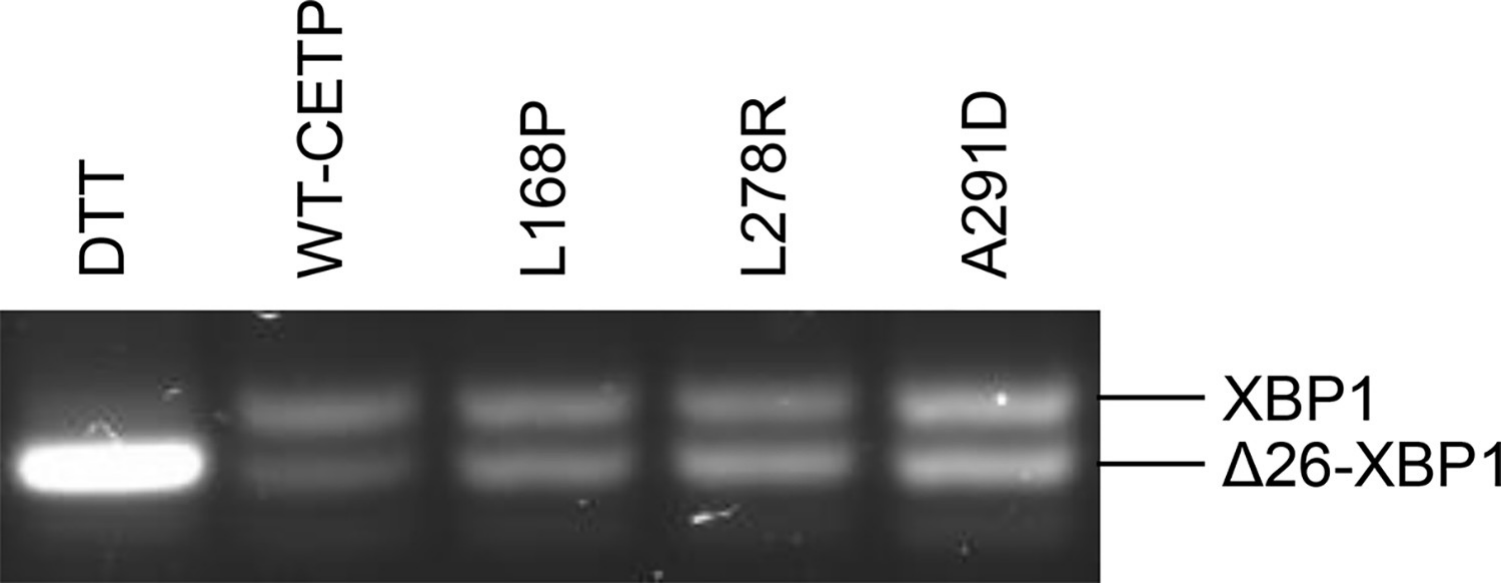
Analysis of ER stress caused by non-secreted CETP variants. HEK293 cells were transiently transfected with the WT-CETP plasmid or with plasmids encoding the non-secreted CETP variants L168P, L278R, or A291D. HEK293 cells transiently transfected with WT-CETP plasmid were incubated with DTT (5 mM) overnight as a positive control for ER stress. mRNA was isolated, reverse transcribed and a relevant part of the XBP1 cDNA was amplified by PCR and subjected to agarose gel electrophoresis. The band representing XBP1 mRNA without skipping of the 26 nucleotides is indicated as XBP1, and the band representing skipping of the 26 nucleotides is indicated as Δ26-XBP1. One representative gel from three separate experiments is shown. ER stress is observed for all of the transfected cells, but to a much lower degree than the positive control. The three non-secreted variants did not cause more ER stress than WT-CETP.

The band size in lysate and the lack of a band in the media for L168P, L278R, and A291D indicate that these variants were translocated into the ER but were not secreted. Possibly, the underlying mechanism could be the failure of these proteins to fold properly in the ER, which could lead to ER stress and proteasomal degradation [37]. The ER stress sensor inositol-requiring enzyme 1 becomes phosphorylated upon ER stress. This activates its endoribonuclease activity and causes the skipping of 26 nucleotides in the mRNA of the *XBP1* gene [38]. Thus, analysis of XBP1 mRNA can be used to identify ER stress. This analysis revealed that ER stress was generated both for WT-CETP and the three non-secreted variants, but to a much lower degree than the positive control (Fig 5).

### Use of chaperones to increase the amounts of secreted CETP

Even though the three non-secreted CETP variants did not cause more ER stress than WT-CETP, our data indicate that the functional consequences of missense variants in the *CETP* gene mainly result from their effects on protein secretion. Thus, studies were undertaken to determine whether the amounts of secreted protein could be increased by the use of chemical chaperones. The chemical chaperones that were studied were 4-PBA, TMAO, glycerol, **and** DMSO. 4-PBA is a hydrophobic chaperone that interacts with exposed hydrophobic segments of an unfolded protein to assist its folding in the ER [39]. TMAO is a cellular osmolyte that promotes the conversion of an unfolded protein to its native state [40]. Glycerol and DMSO sequester water molecules and alter the solvent conditions for the protein [41]. However, none of the four chemical chaperones had any effect on the secretion of these variants (S5 Fig).

## Discussion

In this study, we have examined the functional consequences of 24 missense variants in the *CETP* gene that have been reported to affect levels of HDL cholesterol. For these studies, HEK293 cells have been transiently transfected with the respective CETP missense variant plasmids.

V6D and A15G are localized in the signal peptide sequence, and their protein function depends on whether or not the protein is correctly translocated to the ER. Our data indicate that A15G is mostly translocated correctly. The V6D variant is not recognized by the signal recognition particle, and the protein is translated in the cytosol and therefore not glycosylated in the ER. The 22 other CETP variants were synthesized and translocated into the ER in a normal fashion. Even though there were similar amounts of the different CETP variants in lysates, there was a wide variation in the levels of CETP detected in the media. Other studies on CETP variants have also reported similar findings, with comparable amounts of CETP protein in lysates and large differences in the levels of secreted CETP in the media [16, 22, 26].

A key finding in our study was the statistically significant positive correlation between the amount of secreted protein and the lipid transfer activity of the different CETP variants. Thus, the different variants had specific lipid transfer activities comparable to that of WT-CETP. Similar observations have been made by others [26, 29]. Our data, therefore, indicate that the functional consequences of these missense variants are mediated predominantly by their effects on secretion and not by effects on extracellular lipid transfer activity. As expected, the four non-secreted variants had virtually no lipid transfer activity, and are therefore considered as causes of autosomal dominant hyperalphalipoproteinemia. Our data also indicate that mutant CETP protein found in the media in amounts below a certain threshold, can possibly cause a mild form of autosomal dominant hyperalphalipoproteinemia.

Very little information exists regarding the mechanisms by which the secretion of CETP is regulated and how variants in the *CETP* gene affect secretion. Our findings that the non-secreted variants generated ER stress in a similar fashion to that of WT-CETP indicate that folding defects in the ER followed by proteasomal degradation are not a likely cause of the reduced secretion. Also, Lira et al. [42] report no difference in the levels of ER stress in cells transfected with the WT-CETP plasmid or a CETP plasmid encoding a non-secreted variant. Moreover, chemical chaperones known to assist in proper protein folding in the ER did not promote secretion. One observation that could be of relevance for factors affecting secretion of CETP, is that secreted CETP has been suggested to contain four lipid molecules that have been incorporated intracellularly [12]. One could therefore speculate that variants in the *CETP* gene that prevent proper intracellular lipidation may affect secretion and thereby affect the extracellular lipid transfer activity. In the study of Qiu et al. [12], four of the 14 generated variants affecting residues of the hydrophobic tunnel were not secreted.

Concerning the secretion of the CETP variants, the underlying variants could be categorized into three classes. Variants in Class A (V6D, L168P, L278R, and A291D) that prevented the respective protein from being secreted, are considered loss-of-function variants that cause monogenic hyperalphalipoproteinemia. These four variants affect hydrophobic residues of the protein. The V6D variant, which has a nonpolar, neutral valine replaced with a polar acidic aspartic acid in the middle of the signal peptide, was not translocated to the ER and was therefore not secreted. This observation is in agreement with a previous report [16]. Both Nagano et al. [22] and Thompson et al. [26] have found that L168P is not secreted. p.Leu168 is not evolutionarily conserved (S4 Fig), but introducing a proline at this position could make the backbone less flexible and affect protein folding. Ohtani et al. [23] have found that L278R is not secreted. p.Leu278 is highly conserved and contributes to the lining of the hydrophobic tunnel. Introducing an arginine at this position is assumed to affect the protein structure and interfere with the incorporation of lipids in the tunnel. To our knowledge, A291D has not previously been subject to functional characterization. The variant was identified in a patient with an HDL cholesterol level >99^th^ percentile [24]. Residue p.291 is hydrophobic in all vertebrates and is part of the wall of the hydrophobic tunnel. Introducing an acidic aspartate at p.291 is assumed to affect the protein structure and interfere with the incorporation of lipids in the tunnel. Moreover, also A291G was poorly secreted. These data may therefore indicate a key role for p.Ala291 for the secretion of CETP.

Variants in Class B (A291G, L313Q, Y378C, E443K, and D459G) resulted in proteins that were found in very low amounts in the media. A291G is assumed to have a slightly less severe effect on the structure of the hydrophobic tunnel than A291D. However, no vertebrate CETP has glycine at this position (S4 Fig). Regarding L313Q, Thompson et al. [28] have found markedly reduced amounts of protein in the media which again is consistent with our data. In the study of Qiu et al. [12] the L313Q variant had a lipid transfer activity that was approximately 40% of that of WT-CETP. Residue p.313 is conserved as being hydrophobic in all vertebrates which may indicate its importance for lipid transport. As for Y378C, Lloyd et al. [16] have found that this variant was secreted to a much lower extent than that of WT-CETP. Residue p.378 can be found as tyrosine, histidine, phenylalanine, or leucine, and is located at the tip of the C-terminal TULIP domain and packing with other hydrophobic residues. Substitution to a relatively polar cysteine will likely destabilize the protein tip and affect lipid transport. The Class B variant D459G has been extensively studied. It is the most common variant causing hyperalphalipoproteinemia in the Japanese population [29, 43]. However, the resulting phenotype is milder than that of c.1321+1G>A which destroys the donor splice site in intron 14 in the *CETP* gene [7, 44]. Thompson et al. [26] found that the amount of D459G protein secreted was less than 10% of that of WT-CETP, which is similar to our finding. Based on the data documenting that D459G causes a relatively mild form of autosomal dominant hyperalphalipoproteinemia, it is reasonable to conclude that also the other four variants in Class B cause a mild form of hyperalphalipoproteinemia. To our knowledge, E443K has not previously been subject to functional studies. However, substituting a basic lysine for an acidic glutamate at this position might destabilize the protein structure and prevent proper folding and function.

Of the 15 variants in Class C that were secreted in reasonable amounts, a few have previously been subject to functional characterization and the results from these studies are generally in agreement with our data. Lloyd et al. [16] found that R154W lead to reduced secretion of CETP compared to WT-CETP, but had a near-normal lipid transfer activity. In the study of Pirim et al. [19] R154W had similar allele frequencies among African Black individuals with high HDL cholesterol levels or low HDL cholesterol levels. This finding supports the notion that R154W is a neutral genetic variant. Nagano et al. [22] found that the amount of secreted R299C was approximately 40% of that of WT-CETP and that the lipid transfer activity in the medium was approximately 43% of that of WT-CETP. Thompson et al. [28] found markedly reduced levels of the S349Y variant in the media. Tsai et al. [45] reported that A390P and R468Q were found in higher amounts in human plasma than WT-CETP. In our system, the measured lipid transfer activity of A390P is approximately double of that of WT-CETP. However, the specific lipid transfer activity of A390P was close to that of WT-CETP. A390P has been found both in patients with low and high HDL cholesterol levels [19]. Thus, it is unclear whether A390P is a neutral genetic variant. The finding that residues p.Thr61, p.Arg154, p.Ser221, p.Ser268, p.Ser452 and p.Arg468 are not evolutionarily conserved (S4 Fig) supports the notion that missense variants involving these residues are likely to be neutral genetic variants.

Based on our data and those of others, it seems reasonable to believe that most of the 15 secreted CETP missense variants in Class C do not cause autosomal dominant hyperalphalipoproteinemia or hypoalphalipoproteinemia. Moreover, none of the Class C variants were predicted to be pathogenic or likely pathogenic by applying the ACMG criteria for assessing the pathogenicity of variants [17]. It should also be kept in mind that the vast amounts of data reporting an association between Class C variants and abnormal HDL cholesterol levels have come from genome-wide association studies or resequencing studies of the *CETP* gene in groups of patients with abnormal HDL cholesterol levels. However, care should be exerted when interpreting data from such studies [46–48].

Even though the 15 Class C variants may not cause a monogenic trait, they could nevertheless have a small impact on plasma HDL cholesterol levels that could result from minor effects on the secretion of CETP. However, from clinical studies in humans, there is a controversy about whether there is a correlation between plasma levels of CETP and plasma levels of HDL cholesterol. A negative correlation between the two parameters has been found in some clinical studies [49–53], but not in others [54–59]. Thus, whether the Class C variants could have minor effects on plasma HDL cholesterol levels because of minor changes in plasma concentrations must be considered an unresolved question.

In our study, CETP was overexpressed in a cell system. We recognize this as a potential technical limitation. Overexpressing proteins can sometimes lead to non-physiological interactions or may mask subtler effects seen at physiological expression levels. Moreover, very high amounts of CETP could have influenced the protein’s activity, localization, or other attributes in ways that might not be reflective of its behavior in a natural setting. It is essential to bear in mind that while our findings provide valuable insights, extrapolating these results to in vivo systems or physiological conditions necessitates caution. In future work, examining CETP expression at more physiological levels and in various contexts could shed further light on its functional nuances.

In conclusion, our data indicate that the functional consequences of missense variants in the *CETP* gene are mediated predominantly by their effects on secretion and not by effects on extracellular lipid transfer activity. Class A variants that prevented secretion of the respective protein, cause autosomal dominant hyperalphalipoproteinemia. Class B variants that markedly reduced the levels of secreted protein, cause a mild form of autosomal dominant hyperalphalipoproteinemia. Class C variants which had relatively small effects on the amounts of secreted protein, do not have any clinically relevant effects on HDL cholesterol levels. Our data on how variants in the *CETP* gene affect its secretion, should form the basis for further studies to identify the mechanisms that regulate the secretion and thus extracellular lipid transfer activity of CETP.

## Supporting information

S1 Table Oligonucleotides used for CETP cloning and mutagenesis. The amplification oligos used for cDNA cloning is shown, with CETP start codon in bold in the forward (5’) primer. The reverse (3’) oligo removes the stop codon, and mutated bases are underlined. The forward oligos used for mutagenesis of the WT-CETP plasmid are shown with mutated nucleotides underlined for the respective variants.
(PDF)

S2 Table Means, standard deviations, and p-values for the lipid transfer activities, amounts of protein in media and lysates, and relative CETP mRNA expression for all CETP variants. Variants are normalized to WT-CETP, which was assigned a value of 1.0. The p values were determined using an F-test followed by a two-tailed t-test of two samples assuming equal or unequal variance, depending on the result of the F-test (see the Methods section in the main text). The data shown are from three separate experiments.
(DOCX)

S1 Fig Lipid transfer activity and protein secretion compared in the two CETP variants p.Ile422 and p.Val422. HEK293 cells were transiently transfected with the WT-CETP plasmid or the I422V plasmid. (A) The lipid transfer activity in the media of transfected cells was determined. In order to account for the number of cells in the lysate secreting CETP, lipid transfer activity values were corrected for the protein concentration of lysates from the respective sample. The WT-CETP was assigned a value of 1.0. Values are shown as means (±SD) of three separate experiments. The difference in lipid transfer activity between the WT-CETP and I422V was not statistically significant (ns). (B) Media and lysates from transfected cells were subjected to Western blot analysis using an anti-V5 antibody directed against a C-terminal V5-tag. β-actin was used as a loading control for lysates. One representative blot from three separate experiments is shown. Values from quantitation of the Western blots of media corrected for the protein concentration of the lysate from the respective samples are shown as means (±SD) of three separate experiments. The WT-CETP was assigned a value of 1.0. The difference in protein secretion between the WT-CETP and I422V was not statistically significant (ns).
(PDF)

S2 Fig Relationship between concentration of CETP protein and lipid transfer activity. Increasing amounts of recombinant CETP (SRP6177; Sigma-Aldrich, St. Louis, MO) in a total volume of 1 μL was used to assess the correlation of lipid transfer activity and CETP protein concentration using the CETP Activity Assay Kit II (ab196995; Abcam, Cambridge, UK). The Pearson correlation coefficient (r) and its associated p value (p) are indicated.
(PDF)

S3 Fig Quantitation of CETP mRNA in lysates of HEK293 cells transiently transfected with the WT-CETP plasmid or CETP missense variant plasmids. HEK293 cells were transiently transfected with the WT-CETP plasmid or the indicated mutant CETP plasmids (solid bars). The amounts of WT or mutant CETP mRNA were determined by qPCR. The hatched bars represent controls. The amount of CETP mRNA in cells transfected with the WT-CETP plasmid was assigned a value of 1.0. The values shown are the mean (±SD) of three separate experiments. Variant R175Q is significantly different from WT-CETP (p<0.05). None of the other mutants were statistically different from WT-CETP. Means, standard deviations and p-values for all the mutants are found in S2 Table.
(PDF)

S4 Fig Evolutionary conservation of CETP with the 24 CETP variants indicated. Multiple sequence alignment of 24 CETP genes from different species. Protein sequences that are homologous to human CETP were obtained from the NCBI RefSeq database resources [1] by employing standard BLAST sequence searching [2]. Only sequences with sequence identity above 50% of that of human CETP were included in the dataset. UniProt database [3] was used to evaluate and ensure good quality of the selected sequences. Multiple sequence alignments were generated in Jalview [4] with the use of MUSCLE [5], and visualized with the Clustal coloring scheme. The genes were arranged by class, and bars representing the degree of conservation were added. The species studied were: *Homo sapiens* (Human), *Pan troglodytes* (Chimpanzee), *Castor canadensis* (The North American beaver), *Cavia porcellus* (The Guinea pig), *Loxodonta Africana* (The African bush elephant), *Trichechus manatus latirostris* (West Indian manatee), *Phascolarctos cinereus* (Koala), *Vombatus ursinus* (The common wombat), *Ornithorhynchus anatinus* (The platypus), *Meleagris gallopavo* (Wild turkey), *Anas platyrhynchos* (Mallard), *Calypte anna* (Anna’s hummingbird), *Aptenodytes patagonicus* (King penguin), *Terrapene carolina triunguis* (The three-toed box turtle), *Pogona vitticeps* (The central bearded dragon), *Zootoca vivipara* (The viviparous lizard), *Xenopus laevis* (The African clawed frog), *Nanorana parkeri* (Mountain slow frog*) Danio rerio* (The zebrafish), *Clupea harengus* (Atlantic herring), *Acipenser ruthenus* (The starlet) *Electrophorus electricus* (Electric eel), *Callorhinchus milii* (The Australian ghostshark) and *Amblyraja radiate* (The thorny skate).
(PDF)

S5 Fig Effect of chemical chaperones on the amounts of CETP secreted. HEK293 cells were transiently transfected with the WT-CETP plasmid or with plasmids encoding the non-secreted CETP variants L168P, L278R or A291D. The transfected cells were cultured overnight with the chemical chaperones TMAO (100 mM), glycerol (2.5%), DMSO (2%) or 4-PBA (5 mM). Western blot analyses were performed on the collected media using an antibody against the C-terminal V5 tag. One representative Western blot from three separate experiments is shown. These data indicate that none of the chemical chaperones increased the amounts of secreted protein for the three non-secreted variants.
(PDF)

S1 Raw images (PDF)
