# Correction: Variants in the *CETP* gene affect levels of HDL cholesterol by reducing the amount, and not the specific lipid transfer activity, of secreted CETP

**DOI:** 10.1371/journal.pone.0332400

**Published:** 2025-09-12

**Authors:** Åsa Schawlann Ølnes, Marianne Teigen, Jon K. Laerdahl, Trond P. Leren, Thea Bismo Strøm, Katrine Bjune

An additional affiliation is missing for the first and second author. Åsa Schawlann Ølnes and Marianne Teigen is also affiliated with the The University of Oslo, Norway.
