# Supplementary material for: Variants in the CETP gene affect levels of HDL cholesterol by reducing the amount, and not the specific lipid transfer activity, of secreted CETP
Source: PLoS One. 2023 Dec 1;18(12):e0294764. doi: 10.1371/journal.pone.0294764 (PMC10691695; doi:10.1371/journal.pone.0294764)
Supplement: S1 Table — The amplification oligos used for cDNA cloning is shown, with CETP start codon in bold in the forward (5’) primer. The reverse (3’) oligo removes the stop codon, and mutated bases are underlined. The forward oligos used for mutagenesis of the WT-CETP plasmid are shown with mutated nucleotides underlined for the respective variants. (PDF) [file pone.0294764.s001.pdf]

| **Oligo** | **Sequence, 5’ to 3’** |
|---|---|
| CETP 5’ | GCCTGATAACC**ATG**CTGGCTGCCACAGTCCTG |
| CETP 3’ | GGAGACTTCGCGCTCAAGCTCTGGAGGAAATC |
| A45V CETP | CACGAGACTGTCAAGGTGATC |
| G331S CETP | AAGAGGTTGTCGGCAGCTTCCCCAGC |
| V340I CETP | CAAGTCACCATCCACTGCCTC |
| E420K CETP | AGCAGCTCCAAGTCCATCCAG |
| V6D CETP | TGGCTGCCACAGACCTGACCCTG |
| A15G CETP | CTGGGCAATGGCCATGCCTGCTCCAAA |
| T61M CETP | CCAGAT TCATGGGCGAGAAG |
| D131N CETP | CAGTCCATTAACTTCGAGATC |
| R154Q CETP | GGTAGAGTGCAGACCGATGCC |
| R154W CETP | TGGTAGAGTGTGGACCGATGC C |
| L168P CETP | CATAAGCTGCCCCTGCATCTCCAA |
| R175Q CETP | CAAGGGGAGCAAGAGCCTGGGT |
| S221R CETP | AGGGCTGCCAGAATCCTTTCA |
| G251V CETP | GGAGTCCCATCACAAGGTTCATTTCATC |
| S268L CETP | CCCACCTTCTTGCCCACACTG |
| L278R CETP | TCCCGCATGCGGTACTTCTGG |
| A291D CETP | CACTCGCTGGACAAGGTAGCT |
| A291G CETP | CACTCGCTGGGCAAGGTAGCT |
| R299C CETP | CAGGATGGCTGCCTCATGCTC |
| L313Q CETP | AAGGCAGTGCAGGAGACCTGG |
| E314K CETP | GCAGTGCTGAAGACCTGGGGC TT |
| S349Y CETP | CCCAAGATCTACTGCCAAAAC |
| Y378C CETP | TCTGTAGCTTGCACATTTGAAGAGG |
| A390P CETP | ACTACCGTCCAGCCCTCCTATTCTAAG |
| E443K CETP | TCTCGGCTCAAGGTAGTGTTT |
| S452G CETP | CTCATGAACGGCAAAGGCGTG |
| D459G CETP | AGCCTCTTCGGCATCATCAAC |
| R468Q CETP | CCCTGAGATTATCACTCAAGATGGCTTCC |
