# Supplementary material for: Variants in the CETP gene affect levels of HDL cholesterol by reducing the amount, and not the specific lipid transfer activity, of secreted CETP
Source: PLoS One. 2023 Dec 1;18(12):e0294764. doi: 10.1371/journal.pone.0294764 (PMC10691695; doi:10.1371/journal.pone.0294764)
Supplement: S2 Table — Variants are normalized to WT-CETP, which was assigned a value of 1.0. The p values were determined using an F-test followed by a two-tailed t-test of two samples assuming equal or unequal variance, depending on the result of the F-test (see the Methods section in the main text). The data shown are from three separate experiments. (DOCX) [file pone.0294764.s002.docx]

| **Mutation (p.)** | **Lipid transfer activity (Fig 1A)** | | |  | **Amount of CETP in the media (Fig 1C)** | | |  | **Amount of CETP in the lysates (Fig 1C)** | | |  | | **Relative CETP mRNA expression (S2 Fig)** | | | |
| --- | --- | --- | --- | --- | --- | --- | --- | --- | --- | --- | --- | --- | --- | --- | --- | --- | --- |
|  | **Mean** | **SD** | **p value** |  | **Mean** | **SD** | **p value** |  | **Mean** | **SD** | **p value** |  | **Mean** | | **SD** | **p value** |  |
| A45V | 0.45 | 0.11 | <.001 |  | 1.38 | 0.65 | 0.415 |  | 1.00 | 0.36 | 0.994 |  | 0.92 | | 0.37 | 0.729 |  |
| G331S | 0.84 | 0.19 | 0.063 |  | 0.79 | 0.33 | 0.140 |  | 0.93 | 0.61 | 0.859 |  | 1.10 | | 0.30 | 0.596 |  |
| V340I | 1.22 | 0.09 | 0.003 |  | 0.87 | 0.17 | 0.236 |  | 0.80 | 0.33 | 0.191 |  | 1.21 | | 0.28 | 0.273 |  |
| E420K | 0.82 | 0.05 | 0.005 |  | 0.82 | 0.09 | 0.079 |  | 0.96 | 0.23 | 0.739 |  | 0.95 | | 0.21 | 0.696 |  |
| V6D | 0.07 | 0.05 | <.001 |  | 0.00 | 0.00 | <.001 |  | 0.03 | 0.03 | <.001 |  | 0.72 | | 0.33 | 0.214 |  |
| A15G | 1.21 | 0.28 | 0.332 |  | 1.66 | 0.92 | 0.342 |  | 1.03 | 0.35 | 0.842 |  | 0.63 | | 0.30 | 0.105 |  |
| T61M | 0.70 | 0.17 | 0.002 |  | 0.89 | 0.10 | 0.270 |  | 1.16 | 0.39 | 0.321 |  | 0.74 | | 0.53 | 0.483 |  |
| D131N | 0.64 | 0.23 | 0.105 |  | 0.64 | 0.12 | 0.004 |  | 0.99 | 0.10 | 0.966 |  | 0.71 | | 0.19 | 0.071 |  |
| R154Q | 0.99 | 0.20 | 0.828 |  | 1.35 | 0.65 | 0.444 |  | 1.22 | 0.47 | 0.498 |  | 0.63 | | 0.29 | 0.096 |  |
| R154W | 0.91 | 0.21 | 0.519 |  | 1.25 | 0.44 | 0.434 |  | 1.17 | 0.19 | 0.164 |  | 1.10 | | 0.41 | 0.712 |  |
| L168P | 0.04 | 0.04 | <.001 |  | 0.00 | 0.00 | <.001 |  | 1.20 | 0.23 | 0.121 |  | 0.87 | | 0.20 | 0.327 |  |
| R175Q | 1.09 | 0.26 | 0.607 |  | 1.87 | 0.77 | 0.185 |  | 0.98 | 0.12 | 0.844 |  | 1.29 | | 0.13 | 0.027 |  |
| S221R | 0.88 | 0.22 | 0.434 |  | 1.33 | 0.30 | 0.025 |  | 0.97 | 0.20 | 0.772 |  | 0.94 | | 0.37 | 0.797 |  |
| G251V | 1.11 | 0.21 | 0.454 |  | 0.93 | 0.28 | 0.602 |  | 0.79 | 0.16 | 0.087 |  | 1.32 | | 0.26 | 0.100 |  |
| S268L | 0.61 | 0.12 | <.001 |  | 0.51 | 0.04 | <.001 |  | 1.18 | 0.64 | 0.672 |  | 1.00 | | 0.35 | 0.987 |  |
| L278R | 0.01 | 0.02 | <.001 |  | 0.00 | 0.00 | <.001 |  | 1.31 | 0.61 | 0.465 |  | 0.94 | | 0.22 | 0.686 |  |
| A291D | 0.02 | 0.02 | <.001 |  | 0.00 | 0.00 | <.001 |  | 1.15 | 0.42 | 0.600 |  | 1.26 | | 0.94 | 0.677 |  |
| A291G | 0.24 | 0.03 | <.001 |  | 0.12 | 0.04 | <.001 |  | 1.12 | 0.30 | 0.403 |  | 1.18 | | 0.53 | 0.624 |  |
| R299C | 0.67 | 0.14 | <.001 |  | 0.42 | 0.09 | <.001 |  | 0.87 | 0.13 | 0.240 |  | 1.10 | | 0.33 | 0.616 |  |
| L313Q | 0.20 | 0.08 | <.001 |  | 0.06 | 0.03 | <.001 |  | 0.80 | 0.05 | 0.075 |  | 1.07 | | 0.64 | 0.874 |  |
| E314K | 0.71 | 0.13 | 0.001 |  | 0.62 | 0.09 | 0.002 |  | 0.65 | 0.17 | 0.011 |  | 1.29 | | 0.60 | 0.490 |  |
| S349Y | 0.50 | 0.07 | <.001 |  | 0.30 | 0.03 | <.001 |  | 0.93 | 0.26 | 0.565 |  | 1.02 | | 0.49 | 0.965 |  |
| Y378C | 0.15 | 0.08 | <.001 |  | 0.05 | 0.04 | <.001 |  | 0.95 | 0.29 | 0.724 |  | 0.89 | | 0.51 | 0.757 |  |
| A390P | 2.28 | 0.50 | 0.046 |  | 3.61 | 0.83 | 0.031 |  | 1.11 | 0.54 | 0.761 |  | 0.87 | | 0.53 | 0.718 |  |
| E443K | 0.18 | 0.06 | <.001 |  | 0.02 | 0.03 | <.001 |  | 0.85 | 0.35 | 0.316 |  | 1.17 | | 0.47 | 0.608 |  |
| S452G | 0.48 | 0.09 | <.001 |  | 0.17 | 0.15 | <.001 |  | 0.96 | 0.25 | 0.745 |  | 1.02 | | 0.53 | 0.952 |  |
| D459G | 0.19 | 0.11 | <.001 |  | 0.07 | 0.06 | <.001 |  | 1.16 | 0.28 | 0.234 |  | 1.65 | | 0.90 | 0.339 |  |
| R468Q | 0.73 | 0.09 | 0.001 |  | 0.48 | 0.09 | <.001 |  | 0.89 | 0.27 | 0.430 |  | 1.18 | | 0.67 | 0.685 |  |
