# Supplementary figures and images for: Variants in the CETP gene affect levels of HDL cholesterol by reducing the amount, and not the specific lipid transfer activity, of secreted CETP
Source: PLoS One. 2023 Dec 1;18(12):e0294764. doi: 10.1371/journal.pone.0294764 (PMC10691695; doi:10.1371/journal.pone.0294764)

**A**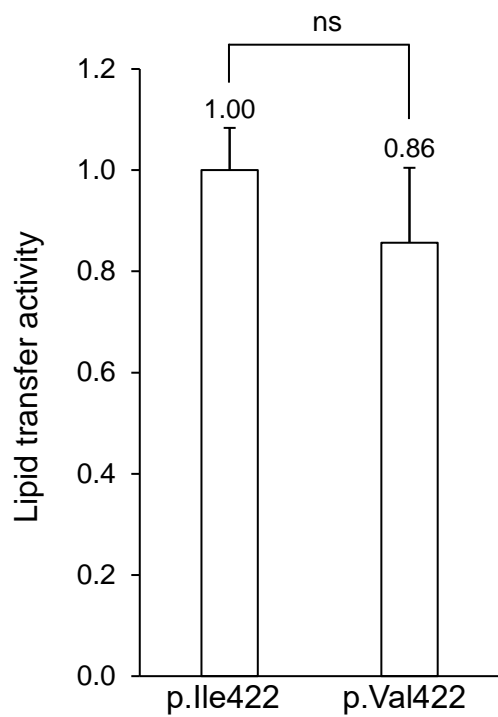**B**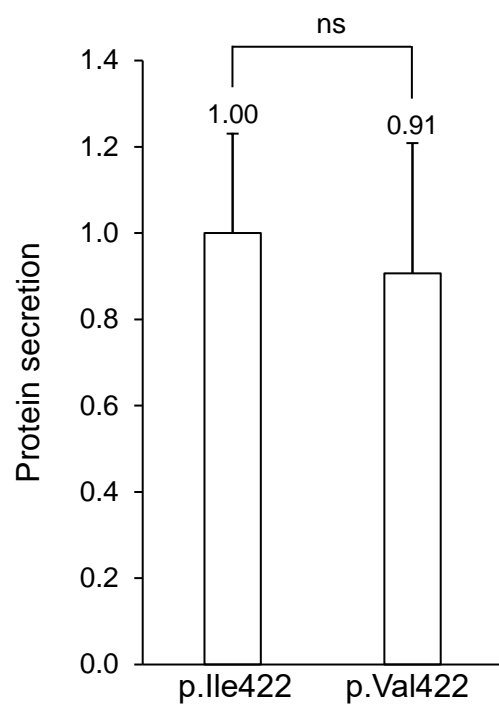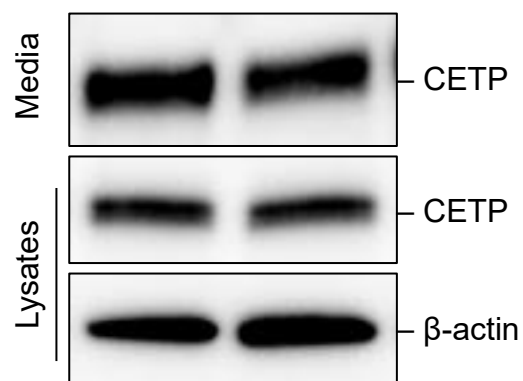

Supplement: S1 Fig — HEK293 cells were transiently transfected with the WT-CETP plasmid or the I422V plasmid. (A) The lipid transfer activity in the media of transfected cells was determined. In order to account for the number of cells in the lysate secreting CETP, lipid transfer activity values were corrected for the protein concentration of lysates from the respective sample. The WT-CETP was assigned a value of 1.0. Values are shown as means (±SD) of three separate experiments. The difference in lipid transfer activity between the WT-CETP and I422V was not statistically significant (ns). (B) Media and lysates from transfected cells were subjected to Western blot analysis using an anti-V5 antibody directed against a C-terminal V5-tag. β-actin was used as a loading control for lysates. One representative blot from three separate experiments is shown. Values from quantitation of the Western blots of media corrected for the protein concentration of the lysate from the respective samples are shown as means (±SD) of three separate experiments. The WT-CETP was assigned a value of 1.0. The difference in protein secretion between the WT-CETP and I422V was not statistically significant (ns). (PDF) [file pone.0294764.s003.pdf]

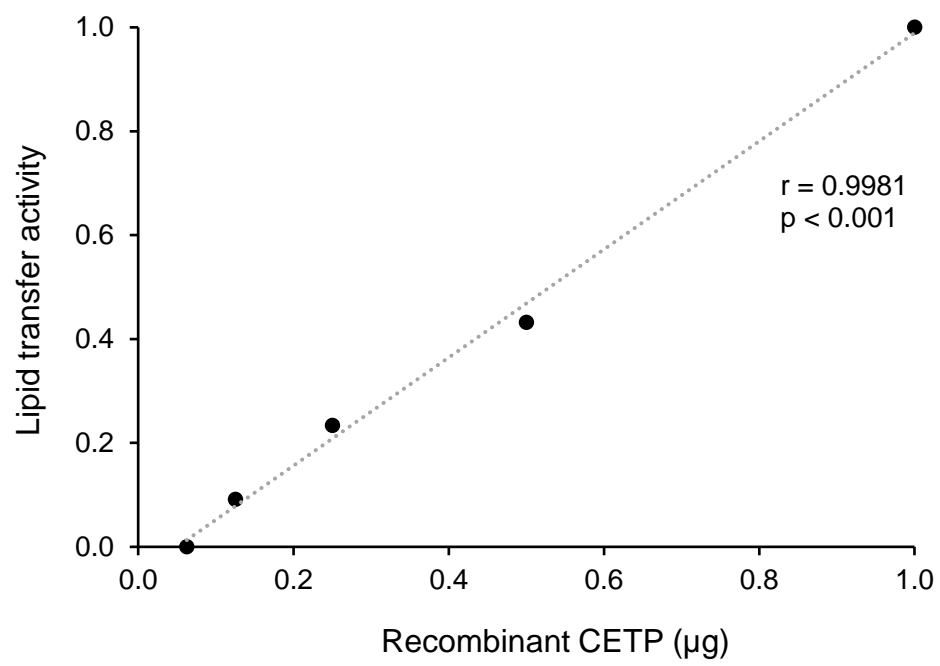

Supplement: S2 Fig — Increasing amounts of recombinant CETP (SRP6177; Sigma-Aldrich, St. Louis, MO) in a total volume of 1 μL was used to assess the correlation of lipid transfer activity and CETP protein concentration using the CETP Activity Assay Kit II (ab196995; Abcam, Cambridge, UK). The Pearson correlation coefficient (r) and its associated p value (p) are indicated. (PDF) [file pone.0294764.s004.pdf]

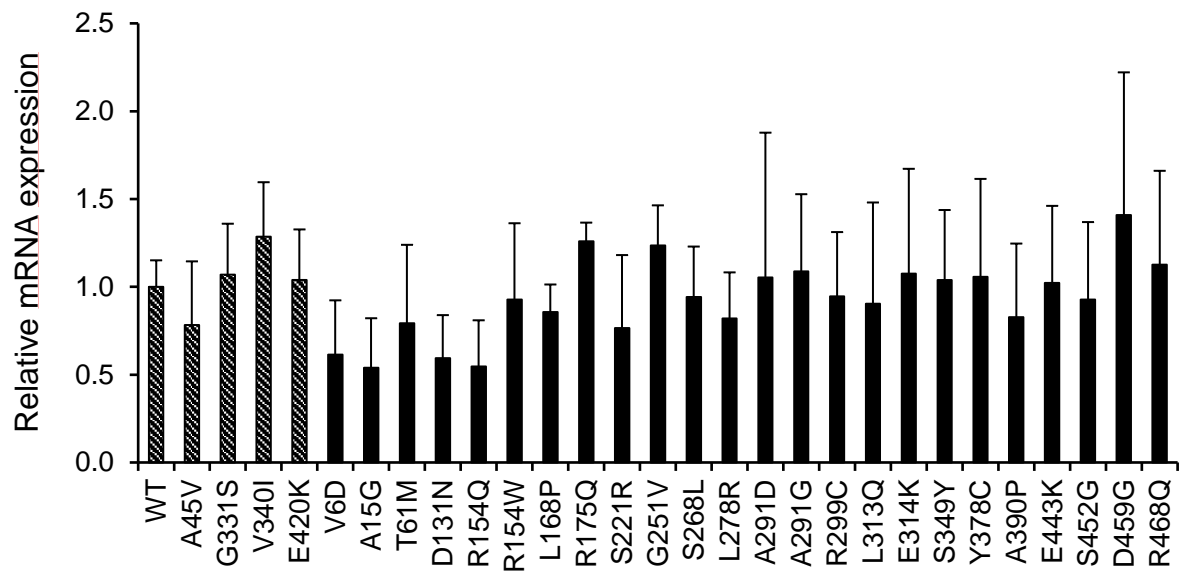

Supplement: S3 Fig — HEK293 cells were transiently transfected with the WT-CETP plasmid or the indicated mutant CETP plasmids (solid bars). The amounts of WT or mutant CETP mRNA were determined by qPCR. The hatched bars represent controls. The amount of CETP mRNA in cells transfected with the WT-CETP plasmid was assigned a value of 1.0. The values shown are the mean (±SD) of three separate experiments. Variant R175Q is significantly different from WT-CETP (p<0.05). None of the other mutants were statistically different from WT-CETP. Means, standard deviations and p-values for all the mutants are found in S2 Table. (PDF) [file pone.0294764.s005.pdf]

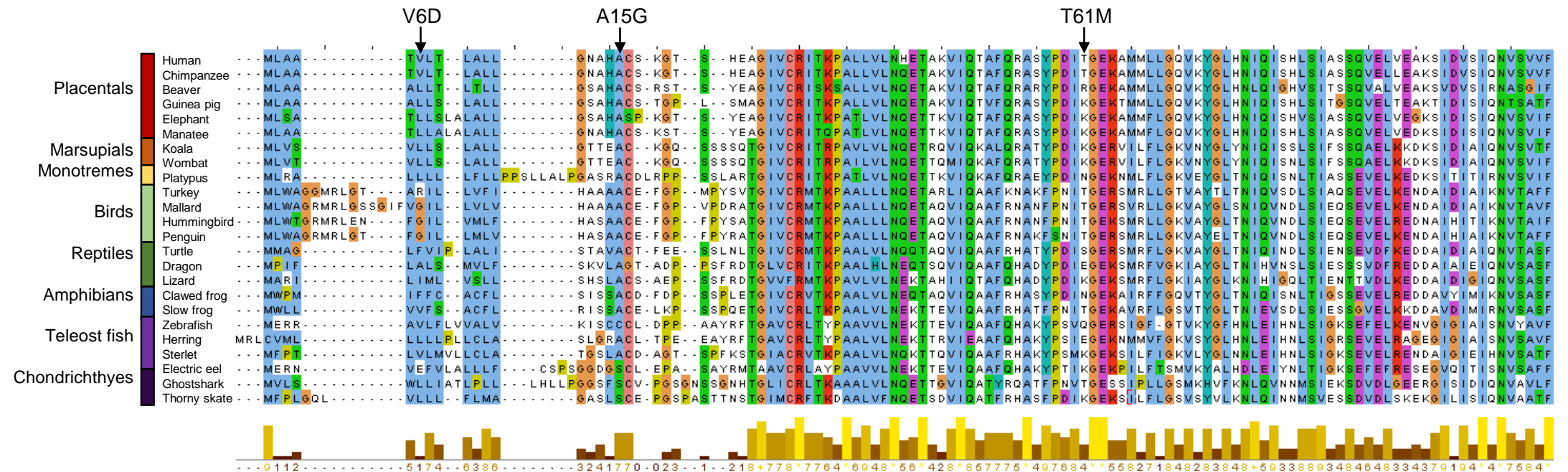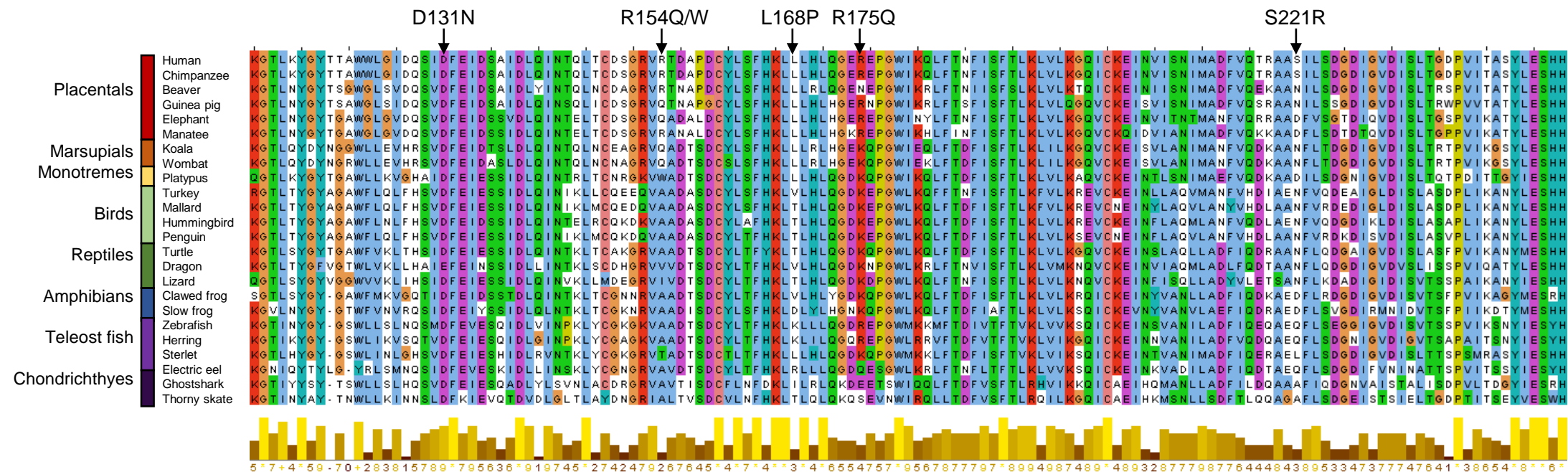

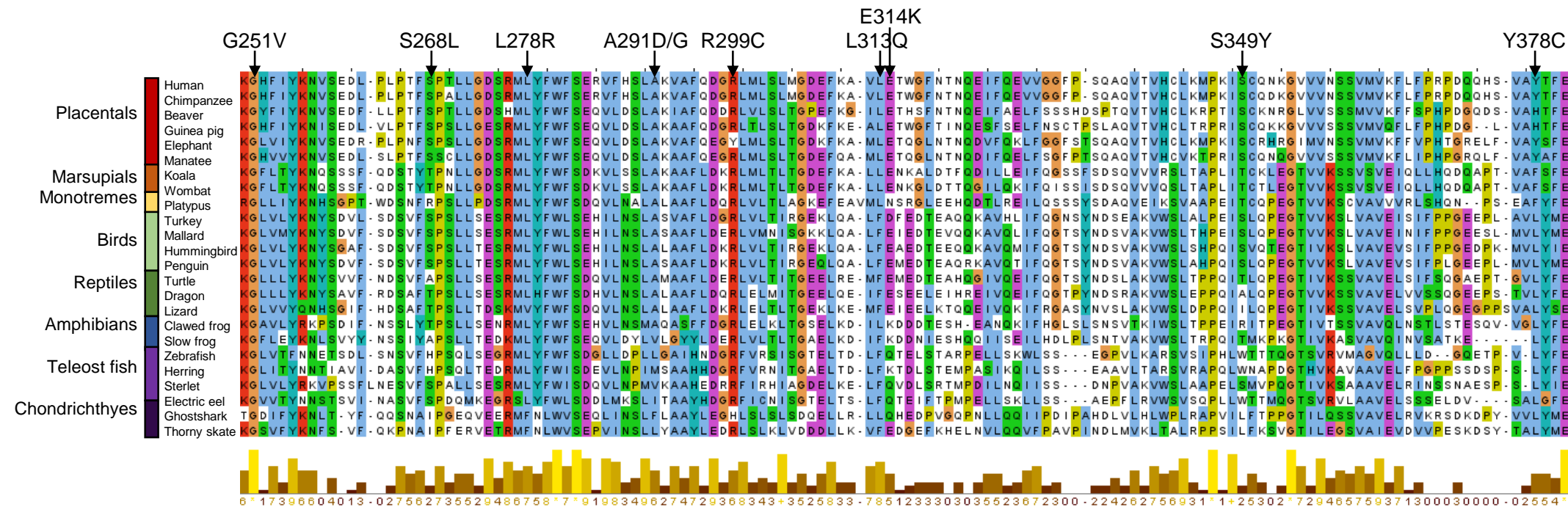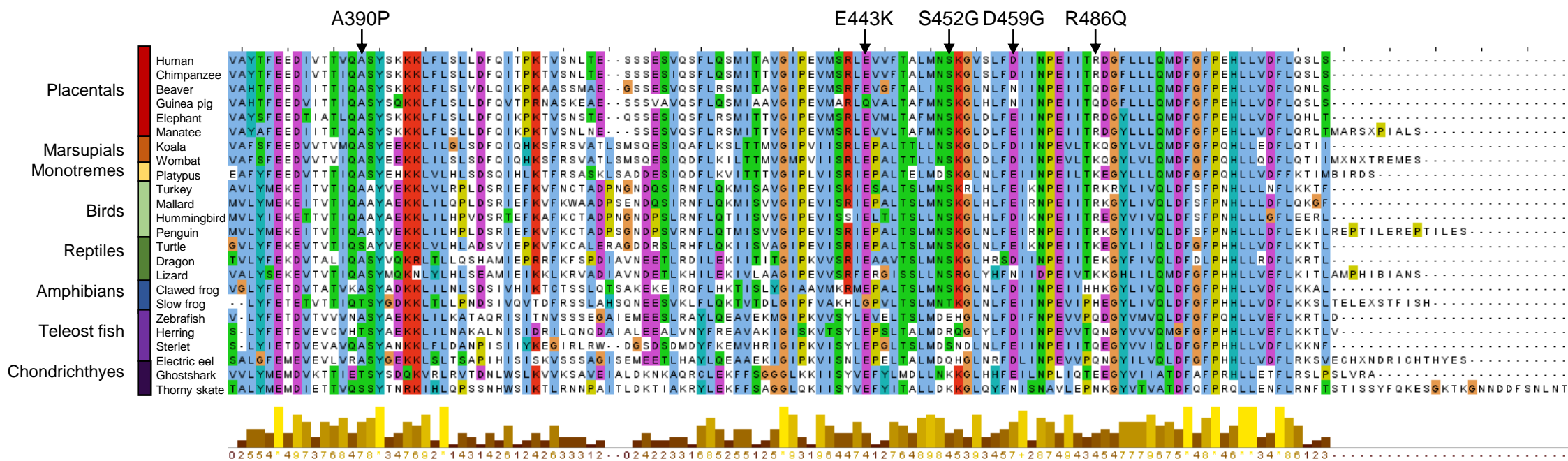

Supplement: S4 Fig — Multiple sequence alignment of 24 CETP genes from different species. Protein sequences that are homologous to human CETP were obtained from the NCBI RefSeq database resources [1] by employing standard BLAST sequence searching [2]. Only sequences with sequence identity above 50% of that of human CETP were included in the dataset. UniProt database [3] was used to evaluate and ensure good quality of the selected sequences. Multiple sequence alignments were generated in Jalview [4] with the use of MUSCLE [5], and visualized with the Clustal coloring scheme. The genes were arranged by class, and bars representing the degree of conservation were added. The species studied were: Homo sapiens (Human), Pan troglodytes (Chimpanzee), Castor canadensis (The North American beaver), Cavia porcellus (The Guinea pig), Loxodonta Africana (The African bush elephant), Trichechus manatus latirostris (West Indian manatee), Phascolarctos cinereus (Koala), Vombatus ursinus (The common wombat), Ornithorhynchus anatinus (The platypus), Meleagris gallopavo (Wild turkey), Anas platyrhynchos (Mallard), Calypte anna (Anna’s hummingbird), Aptenodytes patagonicus (King penguin), Terrapene carolina triunguis (The three-toed box turtle), Pogona vitticeps (The central bearded dragon), Zootoca vivipara (The viviparous lizard), Xenopus laevis (The African clawed frog), Nanorana parkeri (Mountain slow frog) Danio rerio (The zebrafish), Clupea harengus (Atlantic herring), Acipenser ruthenus (The starlet) Electrophorus electricus (Electric eel), Callorhinchus milii (The Australian ghostshark) and Amblyraja radiate (The thorny skate). (PDF) [file pone.0294764.s006.pdf]

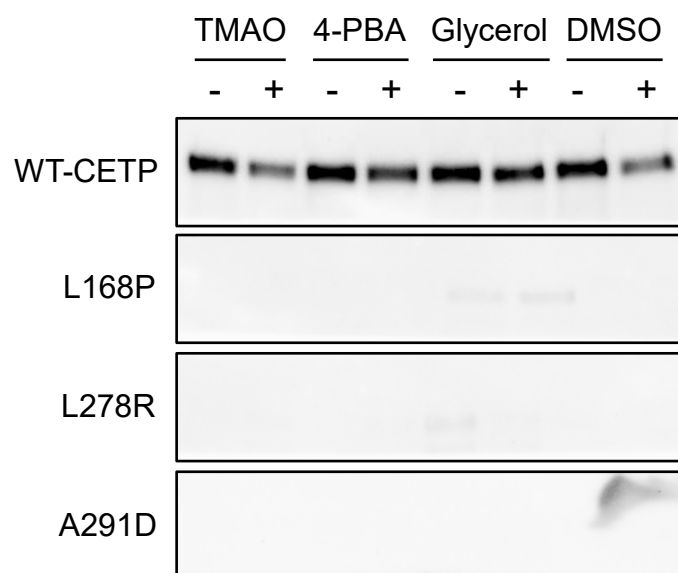

Supplement: S5 Fig — HEK293 cells were transiently transfected with the WT-CETP plasmid or with plasmids encoding the non-secreted CETP variants L168P, L278R or A291D. The transfected cells were cultured overnight with the chemical chaperones TMAO (100 mM), glycerol (2.5%), DMSO (2%) or 4-PBA (5 mM). Western blot analyses were performed on the collected media using an antibody against the C-terminal V5 tag. One representative Western blot from three separate experiments is shown. These data indicate that none of the chemical chaperones increased the amounts of secreted protein for the three non-secreted variants. (PDF) [file pone.0294764.s007.pdf]
