# Supplementary material for: Variants in the CETP gene affect levels of HDL cholesterol by reducing the amount, and not the specific lipid transfer activity, of secreted CETP
Source: PLoS One. 2023 Dec 1;18(12):e0294764. doi: 10.1371/journal.pone.0294764 (PMC10691695; doi:10.1371/journal.pone.0294764)
Supplement: S1 Raw images — (PDF) [file pone.0294764.s008.pdf]

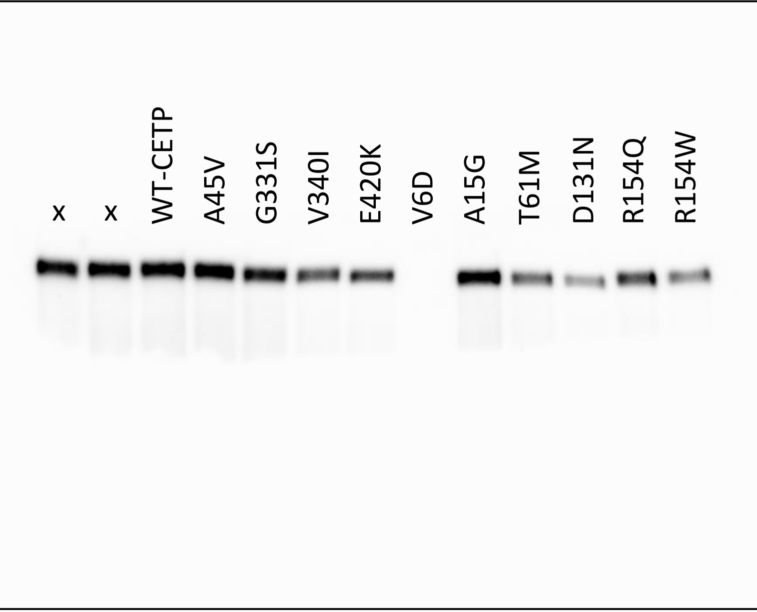

Fig 1: First of three CETP media blots. Chemiluminescence detected on a ChemiDoc Touch Imaging System. Image was analyzed with Image Lab V5.2.1.

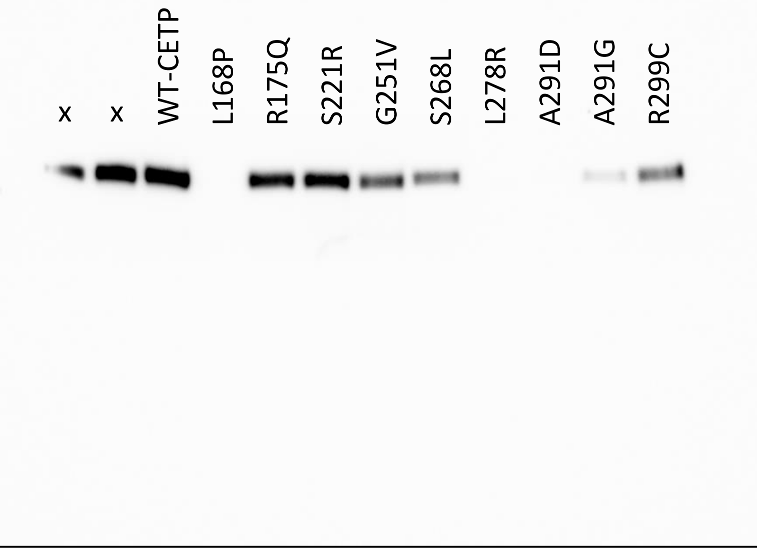

Fig 1: Second of three CETP media blots. Chemiluminescence detected on a ChemiDoc Touch Imaging System. Image was analyzed with Image Lab V5.2.1.

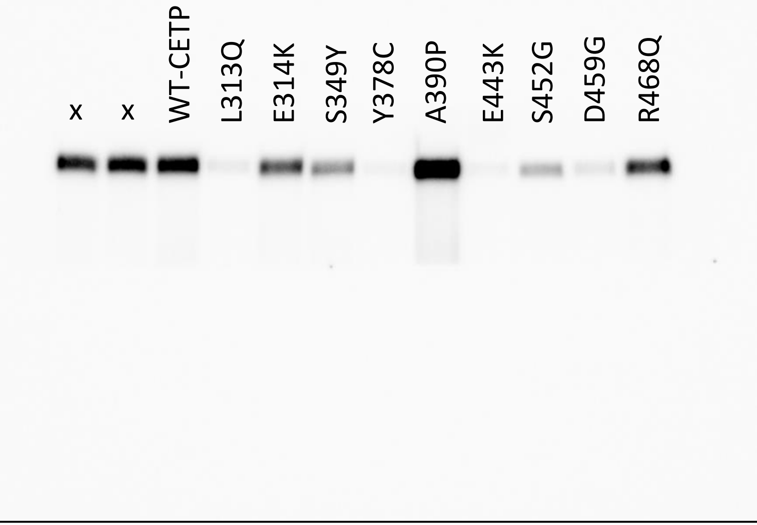

Fig 1: Third of three CETP media blots. Chemiluminescence detected on a ChemiDoc Touch Imaging System. Image was analyzed with Image Lab V5.2.1.

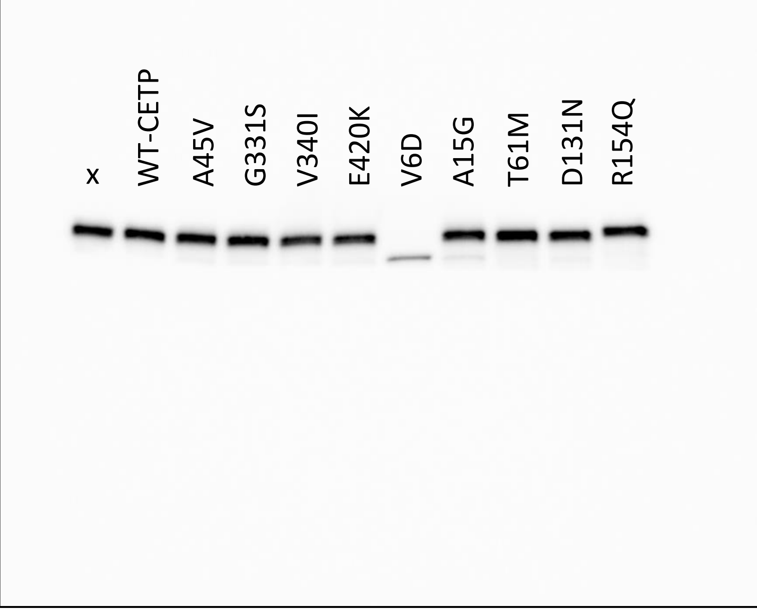

Fig 1: First of three CETP lysate blots. Chemiluminescence detected on a ChemiDoc Touch Imaging System. Image was analyzed with Image Lab V5.2.1.

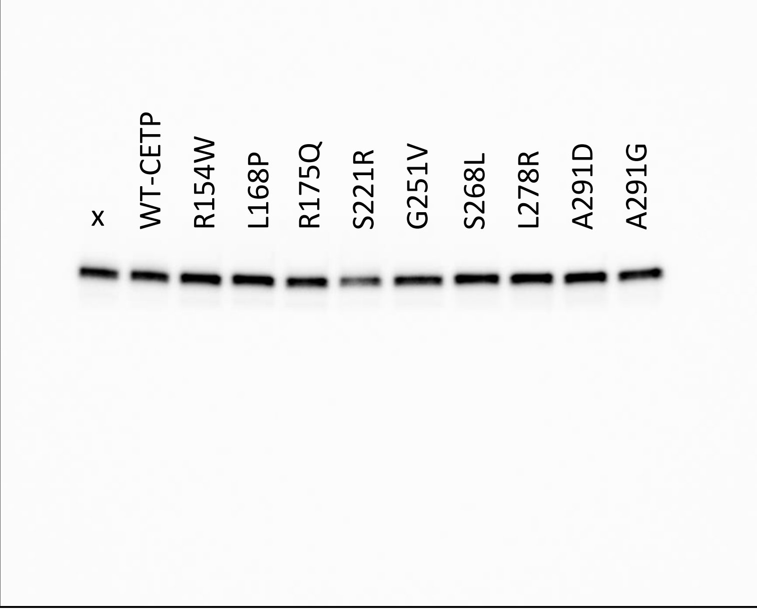

Fig 1: Second of three CETP lysate blots. Chemiluminescence detected on a ChemiDoc Touch Imaging System. Image was analyzed with Image Lab V5.2.1.

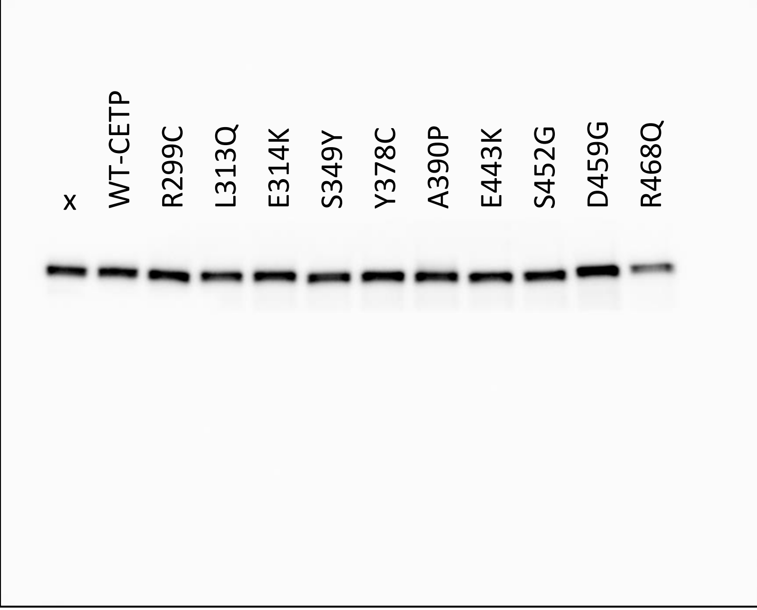

Fig 1: Third of three CETP lysate blots. Chemiluminescence detected on a ChemiDoc Touch Imaging System. Image was analyzed with Image Lab V5.2.1.

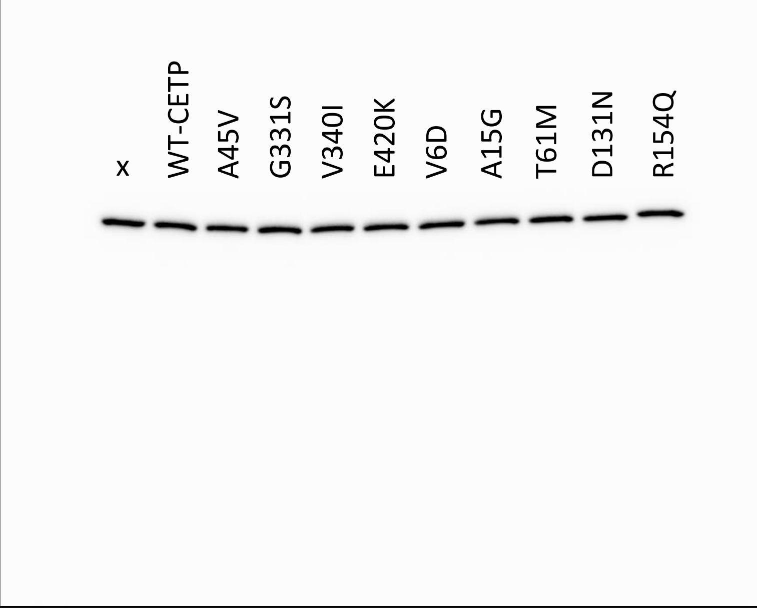

Fig 1: First of three β-actin lysate blots. Chemiluminescence detected on a ChemiDoc Touch Imaging System. Image was analyzed with Image Lab V5.2.1.

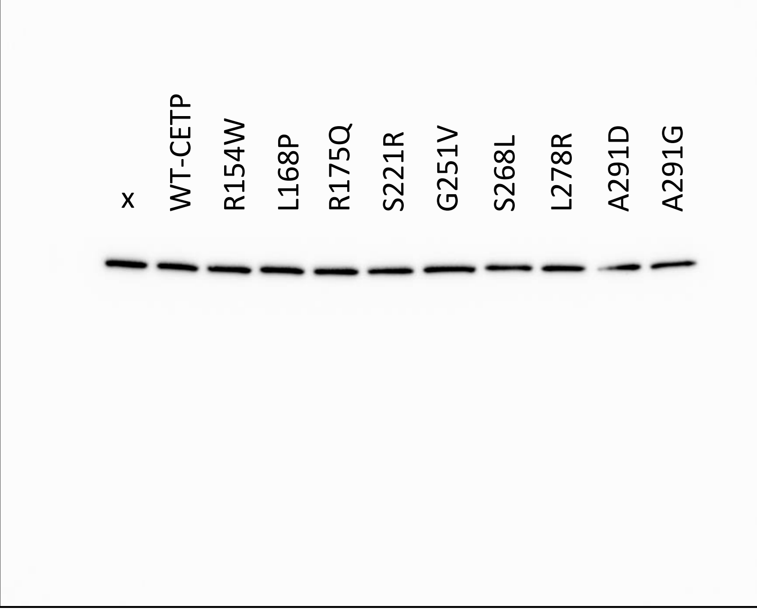

Fig 1: Second of three β-actin lysate blots. Chemiluminescence detected on a ChemiDoc Touch Imaging System. Image was analyzed with Image Lab V5.2.1.

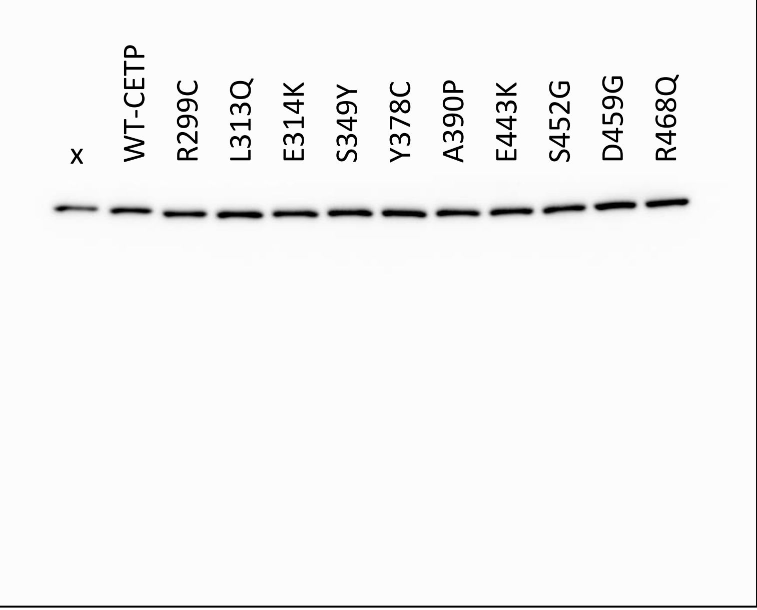

Fig 1: Third of three β-actin lysate blots. Chemiluminescence detected on a ChemiDoc Touch Imaging System. Image was analyzed with Image Lab V5.2.1.

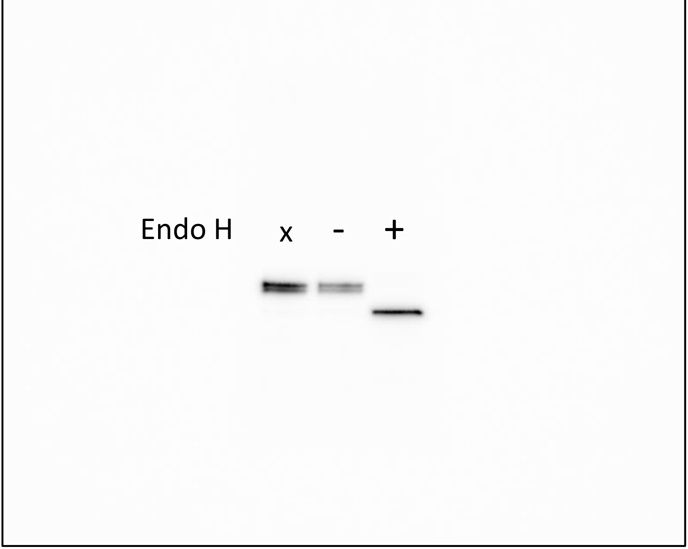

Fig 2: Treatment of WT-CETP with Endo H. Chemiluminescence detected on a ChemiDoc Touch Imaging System. Image was analyzed with Image Lab V5.2.1.

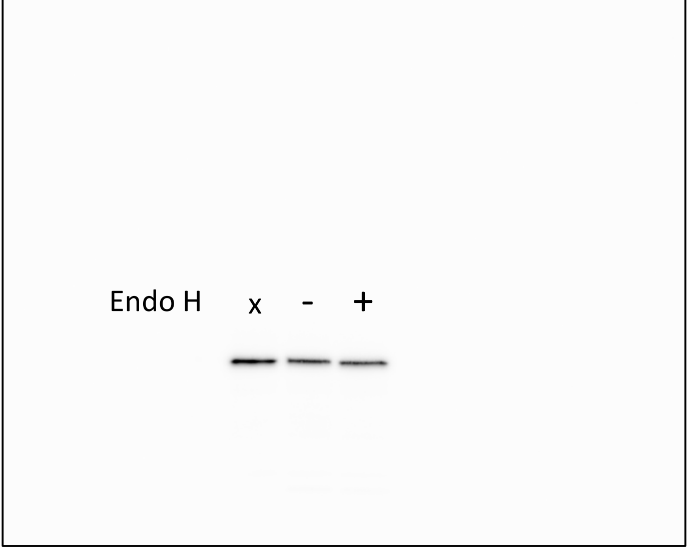

Fig 2: Treatment of V6D with Endo H. Chemiluminescence detected on a ChemiDoc Touch Imaging System. Image was analyzed with Image Lab V5.2.1.

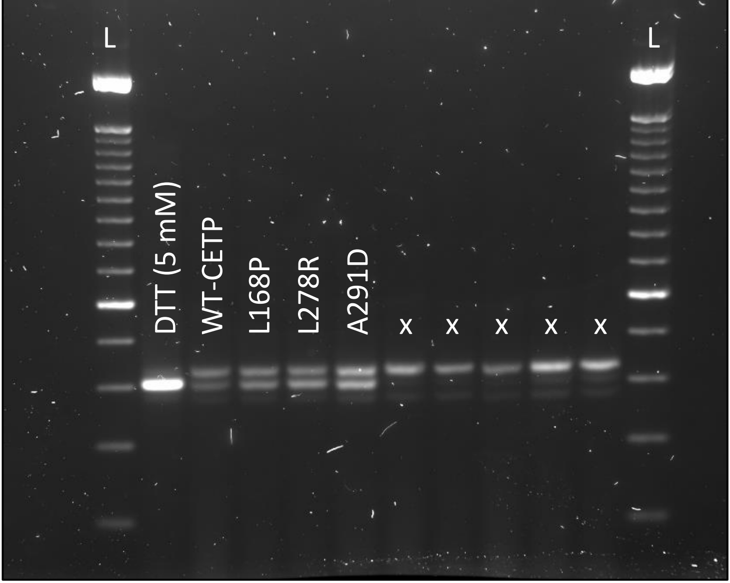

Fig 5: RT-PCR analysis of XBP1 RNA to identify ER stress. RT-PCR products were stained with GelRed Nucleic Acid Stain. TrackIt 100 bp DNA Ladder in first and last well (L). Bands were detected on a ChemiDoc Touch Imaging System. Image was analyzed with Image Lab V5.2.1.

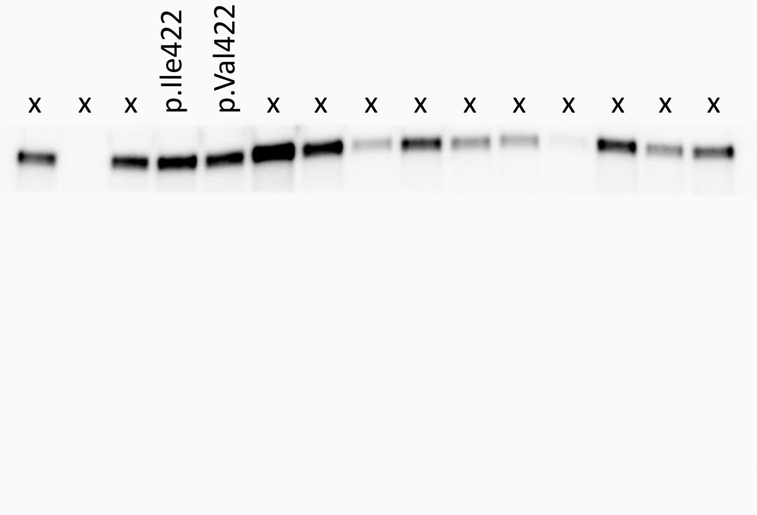

S1 Fig: CETP media blot. Chemiluminescence detected on a ChemiDoc Touch Imaging System. Image was analyzed with Image Lab V5.2.1.

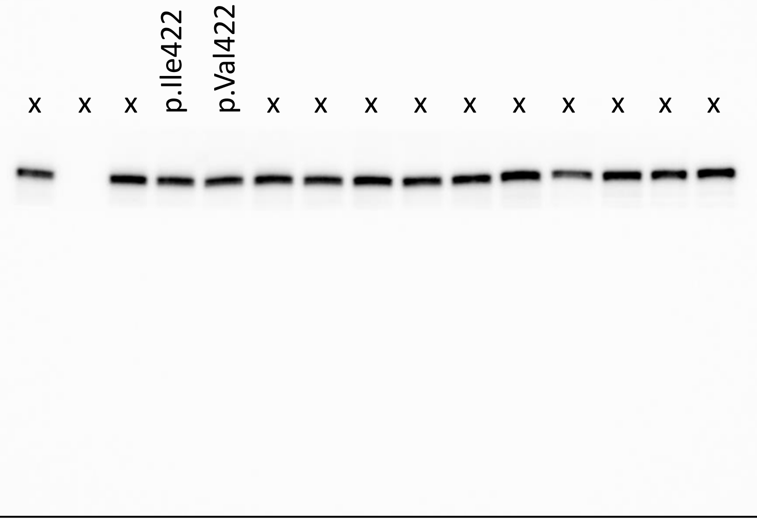

S1 Fig: CETP lysate blot. Chemiluminescence detected on a ChemiDoc Touch Imaging System. Image was analyzed with Image Lab V5.2.1.

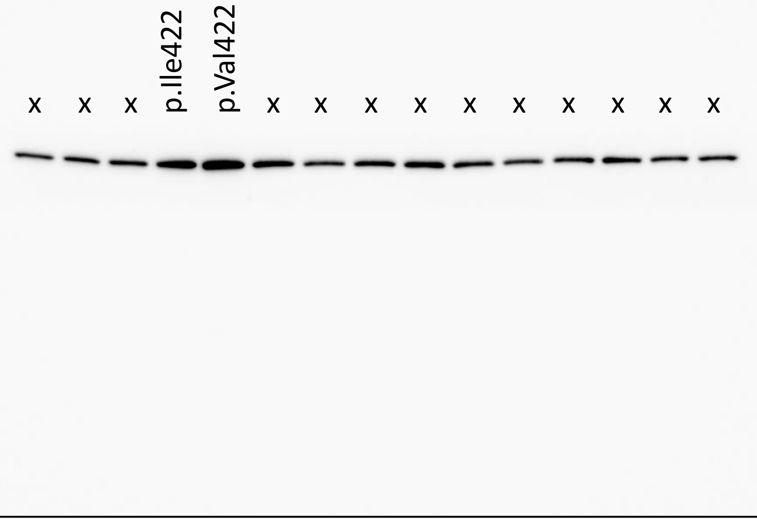

S1 Fig: β-actin lysate blot. Chemiluminescence detected on a ChemiDoc Touch Imaging System. Image was analyzed with Image Lab V5.2.1.

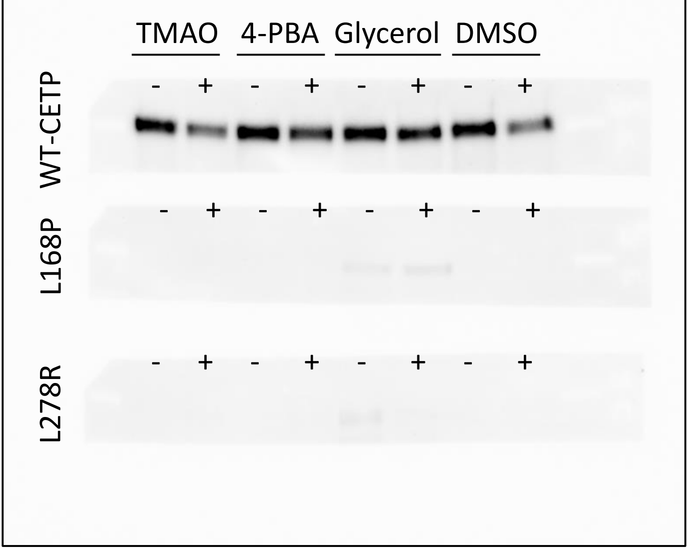

S5 Fig: Treatment of transfected HEK293 cells with chemical chaperones. CETP media blot. Chemiluminescence detected on a ChemiDoc Touch Imaging System. Image was analyzed with Image Lab V5.2.1.

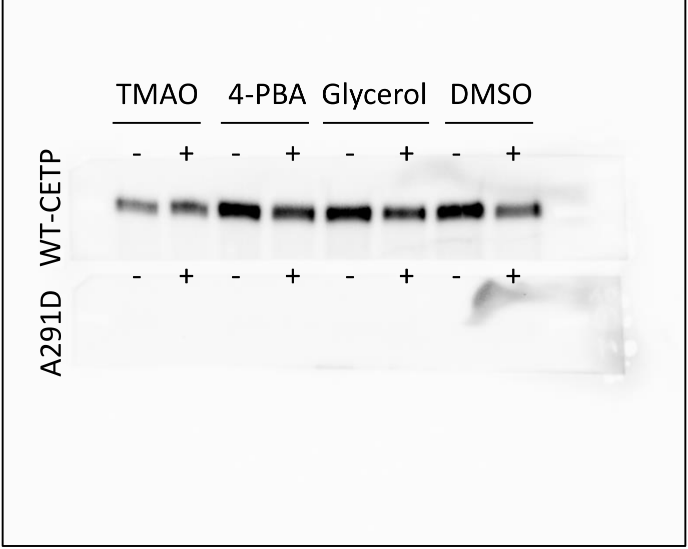

S5 Fig: Treatment of transfected HEK293 cells with chemical chaperones. CETP media blot. Chemiluminescence detected on a ChemiDoc Touch Imaging System. Image was analyzed with Image Lab V5.2.1.
